# Synthesis, Characterisation, and In Vitro Evaluation of Biocompatibility, Antibacterial and Antitumor Activity of Imidazolium Ionic Liquids

**DOI:** 10.3390/pharmaceutics16050642

**Published:** 2024-05-10

**Authors:** Elisabetta Novello, Giuseppina Scalzo, Giovanni D’Agata, Maria G. Raucci, Luigi Ambrosio, Alessandra Soriente, Barbara Tomasello, Cristina Restuccia, Lucia Parafati, Grazia M. L. Consoli, Loredana Ferreri, Antonio Rescifina, Chiara Zagni, Daniela C. Zampino

**Affiliations:** 1 Institute of Polymers, Composites and Biomaterials (IPCB)—CNR, Section of Catania, Via Paolo Gaifami, 18, 95126 Catania, Italy; elisabetta.novello.taa@gmail.com (E.N.); giusyscal88@gmail.com (G.S.); dagata.giovanni@live.com (G.D.); danielaclotilde.zampino@cnr.it (D.C.Z.); 2 Institute of Polymers, Composites and Biomaterials (IPCB)—CNR, Section of Napoli, Viale J.F. Kennedy n.54, Pad.20, 80125 Napoli, Italy; luigi.ambrosio@cnr.it (L.A.); alessandra.soriente@cnr.it (A.S.); 3 Department of Drug and Health Sciences, University of Catania, Viale A. Doria 6, 95125 Catania, Italy; btomase@unict.it (B.T.); arescifina@unict.it (A.R.); 4 Department of Agriculture, Food and Environment, University of Catania, Via Santa Sofia 100, 95123 Catania, Italy; cristina.restuccia@unict.it (C.R.); lucia.parafati@unict.it (L.P.); 5 Institute of Biomolecular Chemistry (ICB)-CNR, via Paolo Gaifami 18, 95126 Catania, Italy; grazia.consoli@icb.cnr.it (G.M.L.C.); loredana.ferreri@cnr.it (L.F.)

**Keywords:** methylimidazolium-based ionic liquids, PVC/IL films, antibacterial tests, in vitro cytotoxicity, antitumor activity

## Abstract

In recent decades, ionic liquids (ILs) have garnered research interest for their noteworthy properties, such as thermal stability, low or no flammability, and negligible vapour pressure. Moreover, their tunability offers limitless opportunities to design ILs with properties suitable for applications in many industrial fields. This study aims to synthetise two series of methylimidazolium ILs bearing long alkyl chain in their cations (C9, C10, C12, C14, C16, C18, C20) and with tetrafluoroborate (BF_4_) and the 1,3-dimethyl-5-sulfoisophthalate (DMSIP) as counter ions. The ILs were characterised using ^1^H-NMR and MALDI-TOF, and their thermal behaviour was investigated through DSC and TGA. Additionally, the antimicrobial, anticancer, and cytotoxic activities of the ILs were analysed. Moreover, the most promising ILs were incorporated at different concentrations (0.5, 1, 5 wt%) into polyvinyl chloride (PVC) by solvent casting to obtain antimicrobial blend films. The thermal properties and stability of the resulting PVC/IL films, along with their hydrophobicity/hydrophilicity, IL surface distribution, and release, were studied using DSC and TGA, contact angle (CA), SEM, and UV–vis spectrometry, respectively. Furthermore, the antimicrobial and cytotoxic properties of blends were analysed. The in vitro results demonstrated that the antimicrobial and antitumor activities of pure ILs against t *Listeria monocytogenes*, *Escherichia coli*, *Pseudomonas fluorescens* strains, and the breast cancer cell line (MCF7), respectively, were mainly dependent on their structure. These activities were higher in the series containing the BF_4_ anion and increased with the increase in the methylimidazolium cation alkyl chain length. However, the elongation of the alkyl chain beyond C16 induced a decrease in antimicrobial activity, indicating a cut-off effect. A similar trend was also observed in terms of in vitro biocompatibility. The loading of both the series of ILs into the PVC matrix did not affect the thermal stability of PVC blend films. However, their T_onset_ decreased with increased IL concentration and alkyl chain length. Similarly, both the series of PVC/IL films became more hydrophilic with increasing IL concentration and alkyl chain. The loading of ILs at 5% concentration led to considerable IL accumulation on the blend film surfaces (as observed in SEM images) and, subsequently, their higher release. The biocompatibility assessment with healthy human dermal fibroblast (HDF) cells and the investigation of antitumoral properties unveiled promising pharmacological characteristics. These findings provide strong support for the potential utilisation of ILs in biomedical applications, especially in the context of cancer therapy and as antibacterial agents to address the challenge of antibiotic resistance. Furthermore, the unique properties of the PVC/IL films make them versatile materials for advancing healthcare technologies, from drug delivery to tissue engineering and antimicrobial coatings to diagnostic devices.

## 1. Introduction

The extreme and often unnecessary use of antibiotics has led to an increasing occurrence of antibiotic resistance phenomena, compromising the efficacy of antibiotics and causing great concern for public health, particularly for hospitalised patients, and inducing bacterial biofilm formation on medical devices and industrial products [1,2]. The massive issue due to the worldwide prevalence of multi-resistant pathogens, the shortage of effective therapies, the lack of successful prevention measures [3], and the residual environmental toxicology of some frequently used chemical biocides [4] have recently stimulated novel research aimed at the design and production of alternative antimicrobial agents. To replace conventional biocides, insure a lower bacterial resistance, and reduce environmental toxicity, new antimicrobial agents, such as essential oils, cationic surfactants, peptides, quaternary ammonium compounds, silver zeolites, ferrocene derivatives, choline-calixarene nano assemblies and ionic liquids (ILs), have been extensively studied [5,6,7,8,9,10,11,12,13,14,15,16,17,18,19]. Moreover, mortality due to the increasing diffusion of tumours and limitations due to anticancer drugs in their treatment still represent serious concerns. In particular, the lack of specificity of most drugs for tumour sites causes severe toxicological effects and the development of drug resistance. Among alternative biocides, ILs, molten salts at a temperature below 100 °C, have attracted significant attention for their interesting features, such as solvation ability, low or no flammability, high electrical conductivity, negligible vapor pressure, thermal stability, low toxicity, etc. [20,21,22]. ILs are mainly composed of large organic cations bearing one or more alkyl substituents and organic/inorganic anions, such as halides, docusate, thiocyanate, tetrafluoroborate, hexafluorophosphate, dicyanammide, etc. [23]. They have been of interest to academic and industrial research, mainly for the planning and development of optimised ILs by varying cations, anions, or both, resulting in many combinations with different chemical, physical, and biological properties. This offers a remarkable gain for their use in precise applications [23].

ILs have been employed to increase reaction rates and selectivity in synthetic chemistry, as solvents in catalytic organic reactions and polymerisation processes to replace conventional organic ones [24,25,26], as safer alternatives for VOCs (Volatile Organic Compounds) in “Green Chemistry” [25,26,27,28,29]. Additionally, they play a role in gas separation and metal extraction as dispersants, surfactants, plasticisers, biosensors, IL-based polymer electrolytes, oxygen transport membranes, porous polymers and polymer gels, nanoparticles, Active Pharmaceutical Ingredients (APIs), antimicrobial, anticancer, and anti-inflammatory agents, as well as a scaffold for biomimetic applications [5,12,20,24,26,30,31,32,33,34,35,36,37].

Various studies reported the antimicrobial activity against different bacteria strains of ILs based on the imidazolium ring [5,6,12,23,37,38,39]. In particular, imidazolium-based ILs with long alkyl chains (11–16 methylene groups) demonstrated intense antimicrobial activity, primarily due to the alkyl chain length in the cation. Indeed, ILs interact with the cell membrane of bacteria, influencing its permeability and resulting in cell death [5,37].

A study reporting the antibacterial mechanism of cationic compounds suggests that it may involve the electrostatic interaction between the phosphate groups of the cell wall and the cationic moieties. Additionally, the hydrophobic segments of these compounds are thought to penetrate the lipid membrane of bacteria, resulting in membrane disruption and subsequent cell death [38].

Ionic liquids (ILs) are increasingly recognized for their potential in antimicrobial applications. Notably, choline-based ILs, particularly those synthesized with choline bicarbonate and geranic acid at ratios of 1:1 and 1:2, have demonstrated important antimicrobial activity [40]. Furthermore, the interaction between ILs and cell membranes has paved the way for exploring combinations of ILs and antibiotics to address infections. Indeed, imidazole-based ILs, when combined with small-molecule antibiotics, have shown synergistic antimicrobial effects against both Gram-positive and Gram-negative bacteria [41]. In a randomized controlled clinical trial conducted by Wu et al. [42], the novel IL-based formulation Ketoconazole-ILs, containing only 1/4 of the Ketoconazole dose found in Daktarin^®^, exhibited superior efficacy and safety in managing tinea pedis, offering a promising treatment option for fungal skin infections. Furthermore, ILs are being investigated for their potential as analgesic agents in managing acute and chronic pain. A clinical study has focused on an ionic liquid analgesic patch developed by MEDRx Co. Ltd. (Higashikagawa, Kagawa, Japan), a Japanese pharmaceutical company, and IL Pharma Inc. (Cambridge, MA, USA). This patch, known as Etodolac with lidocaine, aimed to alleviate back pain and has successfully completed phase III clinical trials. The results highlighted the safety and efficacy of the ILs incorporated into the patch [40].

ILs have been incorporated into various polymeric matrices such as HAPVA, PMMA, PVC, PBT, PC, PET, PLA, Pebax^®^Rnew, SEBS, and more. They serve multiple purposes acting as dispersants [33], plasticisers [43,44,45,46,47], and antimicrobial agents [15,17,19,46,47,48,49,50,51]. Additionally, ILs have been utilised to fabricate IL calcium phosphate-based bionanocomposites [52]. Investigations analysing the use of ILs as potential antitumor agents to replace commonly used drugs aim to develop new antitumour agents capable of decreasing/overcoming the toxicity of chemotherapy and prevent cancer resistance mechanisms [53,54,55,56,57,58,59].

Several studies demonstrated that the chain length of the alkyl substitution at the N-3 position of the imidazolium cation is crucial for the anti-cancer activity of ILs. Compounds with Cl and/or BF_4_ as anion and 1-methy-3-undecylimidazolium as cation showed important activity against different types of cancers [56,60]. Regarding the mechanisms of action, the presence of ILs may compromise the integrity of the cell membrane, followed by changing membrane lipid properties [61].

This study aimed to design a variety of ILs with diverse cation/anion structures for their potential applications as antibacterial and antitumor agents while also considering the assessment of their potential cytotoxicity.

For this purpose, two series of imidazolium-based ILs, featuring a long alkyl chain in their cation (C9, C10, C12, C14, C16, C18, C20) and the tetrafluoroborate (BF_4_) anion along with, for the first time,1,3-dimethyl-5-sulfoisophthalate (DMSIP) as the counterion, were synthetized and evaluated for their antimicrobial and antitumor activity. The DMSIP anion was used for the first time with the cations mentioned above, except for the IL C16mimDMSIP, which has been previously studied [15,17,19]. The prepared ILs were characterised by ^1^H-NMR and MALDI-TOF, and their thermal behaviour was investigated by DSC and TGA. Their antimicrobial, antitumor, and cytotoxic properties were also analysed. To investigate the potential application of the synthesised ILs in the development of antibacterial materials, the most interesting of them were added at different concentrations (0.5, 1, and 5 wt%) to the polyvinyl chloride (PVC), a material broadly used in various industrial fields for its easy workability/processing, compatibility with diverse additives and low cost. This polymer is widely used in building and construction (pipelines, sheaths, gutters, etc.), waterproof clothing, etc. PVC is employed in biomedical device manufacturing due to its biocompatibility and stability. Developing self-disinfectant PVC is crucial for the medical field since it is used to produce several medical and healthcare items (prosthetic limbs, protective films, hearing aids, syringes, tubes, catheters with medical-grade PVC). To achieve this goal, we incorporated ionic liquid into PVC to develop self-disinfectant properties crucial for medical environments. By integrating antimicrobial agents into the polymer matrix, we aim to improve the safety and efficacy of essential medical equipment, including blood containers, catheters, surgical instruments and protective gear [62].

In this study, the PVC/IL film blends were fabricated using the solvent casting method. The goal was to conduct a comprehensive analysis of the physicochemical and biological properties of the blends, with a focus on evaluating their potential applications in the biomedical field. These applications range from drug delivery and tissue engineering to antimicrobial coatings and diagnostic devices. The study involved analysing the blends’ thermal, antimicrobial, and cytotoxic properties, as well as their hydrophobicity/hydrophilicity. Additionally, the distribution of ILs on the film surfaces and their release from the blends were investigated.

## 2. Materials and Methods

### 2.1. Materials

1-Methylimidazole (purity 99%), 1-bromononane (purity 98%), 1-bromodecane (purity 98%), 1-bromododecane (purity 97%), 1-bromotetradecane (purity 97%), 1-bromohexadecane (purity 97%) 1-bromooctadecane (purity ≥ 97%), 1-bromoeicosane (purity ≥ 97%), 1,3-dimethyl-5-sulfoisophthalate sodium salt (purity 98%), dichloromethane (DCM) (purity ≥ 99.9%), tetrafluoroborate sodium salt (purity 98%), anhydrous sodium sulphate (purity 99%), silver nitrate (purity ≥ 99%), ethyl acetate (purity ≥ 99.5%), tetrahydrofuran (THF) (purity 99.9%), dimethyl sulfoxide-*d*_6_ (DMSO-*d*_6_) (99.9 atom % D), trans-2-[3-(4-tert-butylphenyl)-2-methyl-2-propenylidene] malononitrile (DCTB) (purity ≥ 98%) were purchased from Sigma-Aldrich (Milan, Italy) and used as received. The PVC used in this study was plasticised with tris (2-ethylhexyl) trimellitate (TOTM). It was furnished by Consorzio Proplast (Alessandria, Italy) and had a K-value of 65.0, hardness (shore A) of 87.5, and specific gravity of 1.242.

### 2.2. General Description of IL Syntheses

**Scheme 1:**
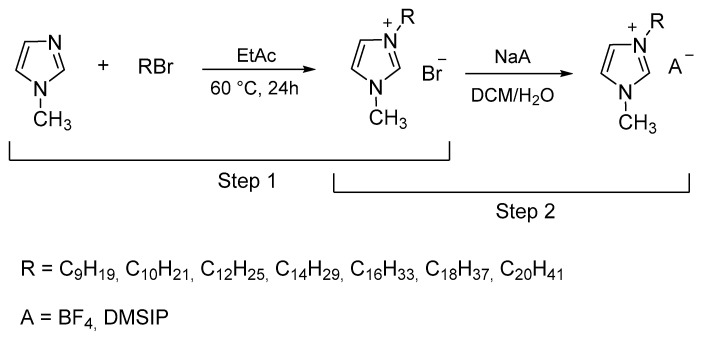
Schematic representation of IL synthesis.

All ILs were synthetised by a two-step method, according to Colonna et al. [48]. These steps involved the alkylation of the methylimidazole ring and metathesis reactions (Scheme 1). The details of the syntheses are provided in the Supplementary Materials data. A brief description of the general synthesis of ILs is reported below.

#### 2.2.1. Alkylation Procedure

The ILs 1-ethyl-3-methylimidazolium bromide (C9mimBr), 1-decyl-3-methylimidazolium bromide (C10mimBr), 1-dodecyl-3-methylimidazolium bromide (C12mimBr), 1-tetradecyl-3-methylimidazolium bromide (C14mimBr), 1-hexadecyl-3-methylimidazolium bromide (C16mimBr), 1-octadecyl-3-methylimidazolium bromide (C18mimBr), 1-eicosyl-3-methylimidazolium bromide (C20mImBr) were synthesised by reacting equimolar amounts of 1-methylimidazole with alkyl bromides containing C9, C10, C12, C14, C16, C18, and C20 hydrocarbon chains, respectively. These reactions were carried out at 60 °C for 24 h under an inert atmosphere with vigorous stirring. The resulting products were washed with ethyl acetate (EtAc) and dried in a vacuum oven for 24 h.

#### 2.2.2. Metathesis Procedure

The ionic exchange between the synthetised 1-alkyl-3-methylimidazolium bromides and the BF_4_ and DMSIP sodium salts was conducted by reacting the solution containing the alkyl-methylimidazolium bromides (0.020 mol) in DCM with the chosen salts (0.021 mol) in water at room temperature (RT) for 1 h. The resulting biphasic solution was then transferred to a separating funnel. Once both phases were clear, well separated, and without precipitates, the organic layer was collected, dried over anhydrous sodium sulphate, and filtrated. The obtained IL powders were then dried in a vacuum drying oven for 48 h at 40–45 °C. The viscous-liquid ILs (C9mimBF_4_, C9mimDMSIP, C10mimBF_4_, C10mimDMSIP) were dried at 25–30 °C for 48 h. To confirm the complete exchange of the bromide counterion, a silver nitrate test was performed on the organic layer. If the exchange was not completed, a new water solution containing the same sodium salt was added to the organic phase to ensure a complete exchange.

### 2.3. Blend Film Preparation

Film preparation was conducted through solvent casting, using PVC pellets and IL powders dried under vacuum for 24 h at 50 °C. The PVC pellets were dissolved in THF (10 g/100 mL concentration) under stirring at RT for 4–5 h. The ILs were then added to the PVC solution at different amounts (0.5, 1, 5 wt%). The resulting solutions were cast on glass plates and left overnight at RT to allow solvent evaporation. The produced films were 100–120 µm thick and were stored under vacuum to prevent moisture adsorption.

### 2.4. Characterisation

#### 2.4.1. Nuclear Magnetic Resonance (NMR) Spectroscopy

A Bruker Avance^TM^ 400 spectrometer and the TOPSPIN 2.1 acquisition software (Billerica, MA, USA) were used to acquire the ^1^H-NMR spectra of the IL samples (10 mg/mL, DMSO-*d*_6_, 20 °C).

#### 2.4.2. Matrix-Assisted Laser Desorption Time of Flight Mass Spectrometry (MALDI-TOF MS) Analysis

The MALDI-TOF analysis was performed using a 4800 MALDI TOF/TOF™ Analyzer (Applied Biosystem, Framingham, MA, USA). The instrument was equipped with a Nd:YAG laser (wavelength of 355 nm) of <500 ps pulse and 200 Hz repetition rate and working in positive-ion mode. All measurements were performed in reflector mode. The mass resolution and accuracy of the MALDI spectra for masses in the range *m*/*z* 200–1000 Da were about 10.000 (full width at half maximum, FWHM) and 1–10 ppm, respectively. Samples were prepared by dissolving the ILs (10 mg/mL) and the matrix trans-2-[3-(4-tert-butylphenyl)-2-methyl-2-propenyldene] malononitrile (0.1 mmol) in THF. The IL solutions were mixed with the matrix solution at sample/matrix ratios (*v*/*v*) of 1:1, 1:2, and 2:1. A total of 1 µL of each sample mixture was spotted onto a MALDI plate holder and held at RT until samples and matrix crystallised. The structural identification of the compounds was made by comparing the MALDI isotopic mass distribution and the peak intensities with the ones calculated.

#### 2.4.3. Thermal Analysis: Differential Scanning Calorimetry (DSC) and Thermogravimetric Analysis (TGA)

DSC measurements of ILs were carried out using the TA Instruments Q100 DSC calorimeter (Milan, Italy), equipped with a liquid sub-ambient accessory. High purity standards (indium and cyclohexane) were used for instrument calibration. The sample (about 5 mg) was sealed in an aluminium pan, equilibrated at −90 °C, heated from −90 to 200 °C (first heating cycle), cooled from 200 to −90 °C (cooling cycle), and heated again from −90 to 200 °C (second heating cycle) at a rate of 10 °C min^−1^ under a flow of nitrogen.

Thermal stability and kinetic parameters of the synthesised ILs were investigated using the TA instruments Q500 thermogravimetric analyser (Milan, Italy). A certain amount of sample (4–5 mg) was placed in a platinum pan and heated at 10 °C min^−1^ from 40 to 600 °C under a flow of nitrogen (60 mL/min).

#### 2.4.4. Scanning Electron Microscopy (SEM)

A Scanning Electron Microscopy (Thermo Fiscer Scienfitic, San Jose, CA, USA) with an integrated energy-dispersive X-ray (EDS) detector was used to analyse the surface morphology of neat PVC and PVC blend films loaded with ILs at 0.5, 1, and 5 wt%. SEM analysis of samples was performed by placing them in carbon tapes and drying and sputter-coating them with gold. Sample images were acquired at 15 kV.

#### 2.4.5. Contact Angle (CA)

The contact angle (CA) determination of film samples was carried out by a DATAPHYSICS OCA 15EC (Filderstadt, Germany) apparatus at RT and using water as liquid. A drop (2 µL) of deionised water was allowed to fall on the specimen surface, fine-tuning its volume by the software of the optical tensiometer. CA results are the average values of three measurements on different areas (lateral and central parts) of the specimen. At least three replicates per sample (±2° standard deviation) were analysed.

#### 2.4.6. IL Release from the Blend Films

Rectangular samples of film blends (24 mm^2^, 6.5 mg, 120–150 µm) containing ILs at concentrations of 0.5, 1, and 5 wt% were immersed in 3 mL of PBS and kept at 37 °C for 24 h. A Jasco V-770 UV–vis–NIR spectrophotometer (Jasco Europe S.R.L., Cremella, LC, Italy) was used to determine the release kinetics. Aliquots were collected at specific interval times (1–24 h), and the optical absorption at 210 nm was measured. Experiments were performed in triplicates, and data are presented as the mean ± S.D.

#### 2.4.7. Antibacterial Screening

##### Bacterial Strain Growth

Bacterial strains used in the present study were chosen based on their proven or emerging resistance to conventional antibiotics [63,64,65].

Within the Di3A (Dipartimento di Agricoltura, Alimentazione e Ambiente, University of Catania, Italy) microbial collection, two Gram-negative strains (*Escherichia coli* and *Pseudomonas fluorescens*) and one Gram-positive strain (*Listeria monocytogenes*) were studied in order to define the potential efficacy of ionic liquids in relation to the different structure of the bacterial cell wall.

All strains were routinely maintained at 4 °C on Nutrient Agar (NA, Oxoid, Basingstoke, UK).

##### Antibacterial Tests

Antimicrobial activity against the aforementioned bacterial strains was evaluated using pure ILs and the ILs loaded into PVC polymeric matrices.

For the pure ILs, the minimal inhibitory concentration (MIC) and minimal bactericidal concentration (MBC) were determined in Nutrient Broth (NB, Oxoid, Basingstoke, UK) medium by broth dilution method, according to the Clinical and Laboratory Standards Institute (CLSI) [66].

All the bacterial strains were individually grown for 24 h at 35 or 27 °C for *P. fluorescens*, with shaking. After the incubation period, the cells were collected by centrifugation and re-suspended in sterile NB to obtain a stock culture of 10^9^ CFU/mL.

Tubes containing 5 mL of NB with decreasing concentrations of ILs (1000, 500, 250, 125, 50, 25, 12.5, 5 and 0 µg/mL) were inoculated with each bacterial stock culture at a final concentration of 10^6^ CFU/mL.

Tubes were incubated at 35 or 27 °C in an orbital shaker and evaluated for the viable count of *E. coli*, *P. fluorescens*, and *L. monocytogenes* after 24 h by spread-plating 100 µL of each suspension on Nutrient Agar (NA, Oxoid, Basingstoke, UK) incubated for 24–48 h at 35 or 27 °C.

In this study, MIC and MBC are expressed in both µg/mL and µM to make the comparison with the literature data easy. Each experiment was performed in triplicate.

The potential antimicrobial activity of the PVC-loaded IL films was evaluated using the disk diffusion method, which measures the growth inhibition halo produced by the film laid on a plate of nutritive medium previously inoculated with the target microbial strain.

In detail, an overnight bacterial cell suspension grown in NB (100 µL at a final concentration of 10^6^ CFU/mL) of *E. coli*, *P. fluorescens*, and *L. monocytogenes* was individually spread on the surface of Petri plates containing Nutrient Agar (NA, Oxoid, Basingstoke, UK) medium. The PVC/IL films containing amounts of ILs at 0.5, 1, and 5 wt.% and the neat PVC ones, used as control, were placed on the plate surface and incubated at 35 or 27 °C for 24–48 h. The inhibitory activity of the films against the target strains was evaluated by measuring the size (mm) of the inhibition halo (no bacterial growth) around the film. Each test was carried out in triplicate.

#### 2.4.8. Biocompatibility/Cytotoxicity

##### Cell Cultures

Cytotoxicity studies on IL powders were carried out using human dermal fibroblast (HDF) cells (Sigma-Aldrich) and a human breast cancer cell line (MCF7). In addition, ILs loaded in PVC polymer substrates were tested for their cytocompatibility and adhesion properties on the HDF. HDF cells were cultured in 75 cm^2^ cell culture flask in Dulbecco’s Modified Eagle’s Medium (DMEM) supplemented with 20% Foetal Bovine Serum (FBS), 1% non-essential amino acid (NEAA), antibiotic solution (streptomycin 100 µg/mL and penicillin 100 U/mL, Sigma Aldrich) and 2 mM l-glutamine. MCF7 cells were grown in DMEM with 10% FBS and the same antibiotics as mentioned above. The cells were maintained in a culture at 37 °C, with 5% CO_2_ and 95% humidity to achieve a confluent layer. Control cells were cultured in a complete medium without any compounds in tissue culture plates. Before biological testing, the samples were sterilised using a UV light for 2 h.

##### Cytotoxicity Tests for IL Powders

A direct cytotoxicity test was used to assess the in vitro cytotoxicity of IL powders. IL powders with different alkyl chains were dissolved in DMEM media to obtain solutions at various concentrations (0.5–1–5 and 10 µg/mL) to determine the best formulation.

After 24 h of incubation of IL solutions (200 µL) with seeded cells under specific environmental conditions (T = 37 °C, HR = 95%, CO_2_ = 5%), cell viability was tested using the Alamar blue assay (AbD Serotec, Milan, Italy) following the manufacturer’s instructions.

In summary, each well received a 200 µL aliquot of Alamar Blue^TM^ diluted 1:10 in phenol red-free medium and was incubated for 4 h at 37 °C, 5% CO_2_. Following this, 100 µL of the solution was transferred to a 96-well plate for colorimetric analysis. Wells with no cells were included to account for any background interference from the redox indicator. Optical density was immediately measured using a spectrophotometer (Victor X3, Perkin Elmer, Waltham, MA, USA) at 560 and 595 nm wavelengths. The results were presented as a percentage of cell viability compared to that of the control (cells grown on tissue culture plates).

##### Cytocompatibility Tests for IL-Loaded Polymer Substrates

The cytotoxicity of IL-loaded polymer substrates was assessed using direct and indirect tests, as detailed below.

Indirect test: ISO 10993-5, which provides test procedures to evaluate the in vitro cytotoxicity of medical devices, was employed to test biocompatibility [67]. The elution test was conducted by incubating the material in a sterile DMEM solution (extraction vehicle prepared as described earlier) at a ratio of 0.1 g/1.0 mL at 37 °C (following ISO 10993-12 guidelines) [68]. After 24 h of elution time, the conditioned media (eluants) were collected, and 200 µL were pipetted into a 96-well plate previously seeded with HDF cells at 80% confluence. The plate was incubated for an additional 24 h (exposure time) [69].

Direct test: cells were seeded directly onto the material surface at 10^3^ cells/well density and incubated for 24 h under specific environmental conditions (T = 37 °C, H = 95%, CO_2_ = 5%).

Cell viability was assessed in both cases using the Alamar blue assay, as described earlier. Furthermore, immunofluorescence microscopy was utilised to examine cell-material interactions and their spread using a fluorescent dye. Specifically, HDF cells were labelled with CellTracker^TM^ Red CMTPX (Invitrogen, Milan, Italy) in a 75 cm^2^ cell culture flask, trypsinised, and then seeded onto IL-loaded polymer substrates. The cells were then incubated for 24 h at 37 °C. Subsequently, non-adherent cells were removed by careful washing with a phosphate-buffered solution (PBS; pH = 7.4). The substrates were observed under a JuLI^TM^ Stage microscope after being washed three times with PBS 1× solution and seeded with cells for an additional 24 h.

#### 2.4.9. Statistical Analysis

All quantitative experiments were performed in triplicate, and the results were expressed as mean ± standard deviation (SD). Statistical analysis was undertaken using GraphPad Prism^®^, version 5.00 (GraphPad Software, La Jolla, CA, USA, www.graphpad). Data were analysed using a Student’s *t*-test, a one-way ANOVA, and a Bonferroni post-test (parametric methods). Group differences of *p* < 0.01, *p* < 0.001, and *p* < 0.0001 were considered statistically significant. Samples within each group were randomly and independently selected from their respective conditions. Normality was assessed using a Shapiro–Wilk test and all groups passed the normality test (α = 0.05).

## 3. Results and Discussion

### 3.1. IL Synthesis, Characterisation, and Fabrication of PVC/IL Film Blends

Two series of ILs formed by methylimidazolium cations with varying alkyl substituents and two different counteranions (Table 1) were prepared following the two-step method reported in Scheme 1. The first step, regarding the alkylation and quaternisation of 1-methylimidazole, through S_N_2 substitution of the alkyl halide with the imidazole ring, yielded a series of 1-alkyl-3-methylimidazolium bromides (C9-C20mimBr). The second step, involving a metathesis reaction between DCM solutions of the obtained bromides with water solutions of the inorganic salts NaBF_4_ or NaDMSIP, produced the expected ILs. A more detailed description of the IL synthesis is reported in the Supplementary Materials.

All ILs were characterised by ^1^H-NMR and MALDI-TOF analyses. The characterisation by ^1^H-NMR allowed us to verify the functionalisation of the imidazole ring with the alkyl chains of different lengths. The chemical shift of the two series of ILs synthetized is reported in Table S1.

The analysis of the MALDI-TOF spectra was necessary to establish the success of the synthesis reactions for the series containing the BF_4_ as anion, while in the case of the series containing DMSIP, ion exchange was also confirmed through ^1^H-NMR analysis, displaying the characteristic proton signals of the counter ion. As an example, in Figures S1 and S2, the ^1^H-NMR spectra of C12mimBF_4_ and C12mimDMSIP are reported. Table S2 labels the formulas, as well as the calculated and measured exact masses of the cations and the adducts (species constituted by two cations and the anion) of the ILs produced.

The film preparation was performed using the solvent casting method. The PVC pellets were solubilised in THF at 10 g/100 mL concentration. Only the ILs C12mimBF_4_, C12mimDMSIP, C14mimBF_4_, C14mimDMSIP, C16mimBF_4_, and C16mimDMSIP were added at three different concentrations (0.5, 1, and 5 wt%) to the PVC solution because they showed better antibacterial and antitumor activity than that of the other synthetised ILs. The complete dissolution of the PVC/IL solutions occurred within 4–5 h under stirring at RT. The solutions were then poured onto glass plates and evaporated at RT overnight. To prevent the produced films from absorbing moisture, they were kept under vacuum until characterisation.

In general, the blend films (100–120 µm thickness) were colourless, soft, and transparent, demonstrating a good dispersion of ILs into the PVC matrix. Only the PVC film loaded with the IL C16mimBF_4_ was slightly whitish and opaque (Figure S3F) due to the IL being less miscible with the polymer matrix.

### 3.2. Thermal Analysis of ILs and PVC/IL Blends

The direct inclusion of active agents during polymer processing for industrial product development serves to circumvent complex synthesis procedures. However, this approach requires evaluating the thermal properties and stability of the biocides. This assessment is critical to prevent potential degradation during industrial manufacturing, which could otherwise impact the performance of the materials.

The thermal properties and stability of the neat ILs and PVC/IL blends were analysed using DSC and TGA.

**Figure 1:**
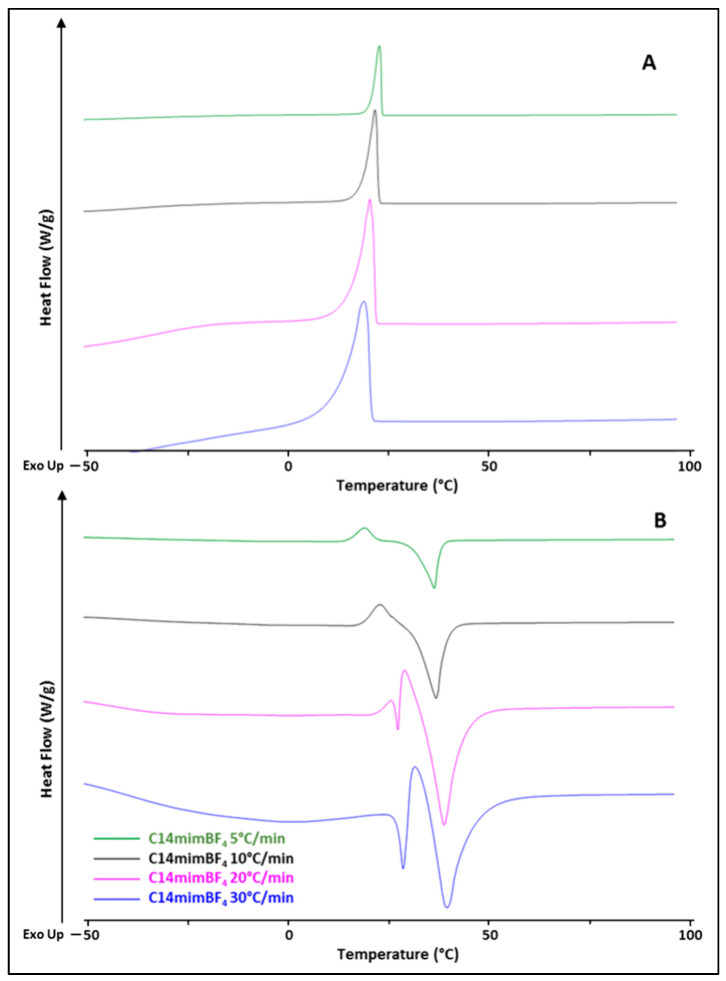
DSC curves of C14mimBF_4_ at different cooling and heating runs. (**A**) Cooling runs; (**B**) second heating runs. Curves are shifted for clarity. The legend refers to both graphics.

DSC measurements were carried out by cooling and heating scans at 10 °C/min from −90 °C to 200 °C. They allowed for identifying temperature transitions. In particular, the glass transition (T_g_), cold crystallisation (T_cc_), and melting (T_m_) temperatures, recorded during the heating scan, are the midpoint of a variation from the amorphous glass state to a liquid state and the onset of exothermic and endothermic peaks, respectively. The crystallisation (T_c_) temperature is an exothermic peak detected during the cooling run. To perform an exhaustive study on the thermal transitions, paying attention to variations in cooling and heating rates is essential. Indeed, under fast rates, the transition peaks may overlap, whereas under slow rates, the peaks appear more defined and separate [70]. Considering the literature data, a series of preliminary DSC measurements at different cooling and heating rates (5, 10, 20, and 30 °C/min) was carried out. Data depicted in Figure 1 are DSC curves of the IL C14mimBF_4_, reported as an example of variations upon different cooling and heating rates. The transition peaks were clearly separated during cooling and second heating runs at 5 and 10 °C/min. Notably, significant shifts in T_c_ and T_m_ temperatures were not observed, indicating slight differences of 1–4 °C due to peak broadening from the increased heating/cooling rates. On the contrary, the T_cc_ peak on the second heating cycle displayed a noticeable shift due to the higher rates. This led to an approach and partial overlap of the T_cc_ and T_m_ peaks, as previously reported [70]. Considering these results and the more common use of the 10 °C/min rate, it was decided to adopt this rate for the subsequent measurements.

**Figure 2:**
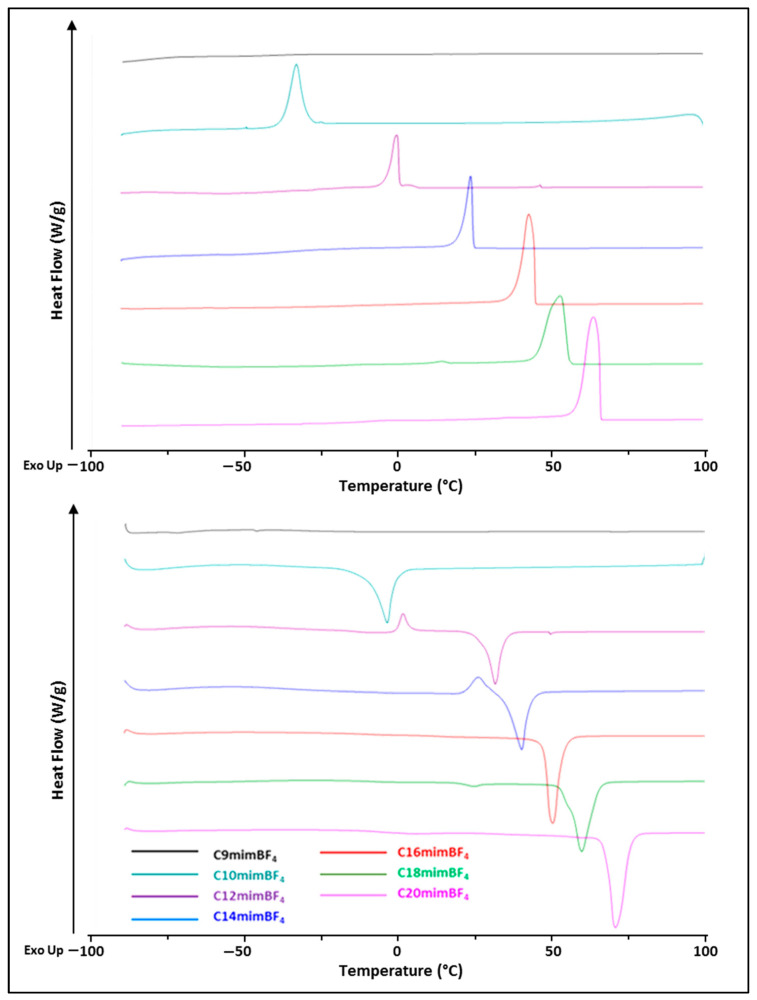
DSC curves of the series CnMimBF_4_ at 10 °C/min cooling and heating runs. (**Up**) cooling cycle; (**Down**) second heating cycle. Curves are displaced for clarity. The legend refers to both graphics.

Previous studies on the thermal properties of ILs grouped them according to three main thermal behaviours [70,71]. The ILs of the first group show freezing and melting transitions on cooling and heating scans, respectively. The ILs C10mimBF_4_, C16mimBF_4_, C18mimBF_4_, and C20mimBF_4_, showing T_c_ values upon cooling of −33, 43, 53, and 64 °C, and T_m_ values on a second heating run of −4, 50, 59 and 70 °C, respectively, display this behaviour (Figure 2).

**Figure 3:**
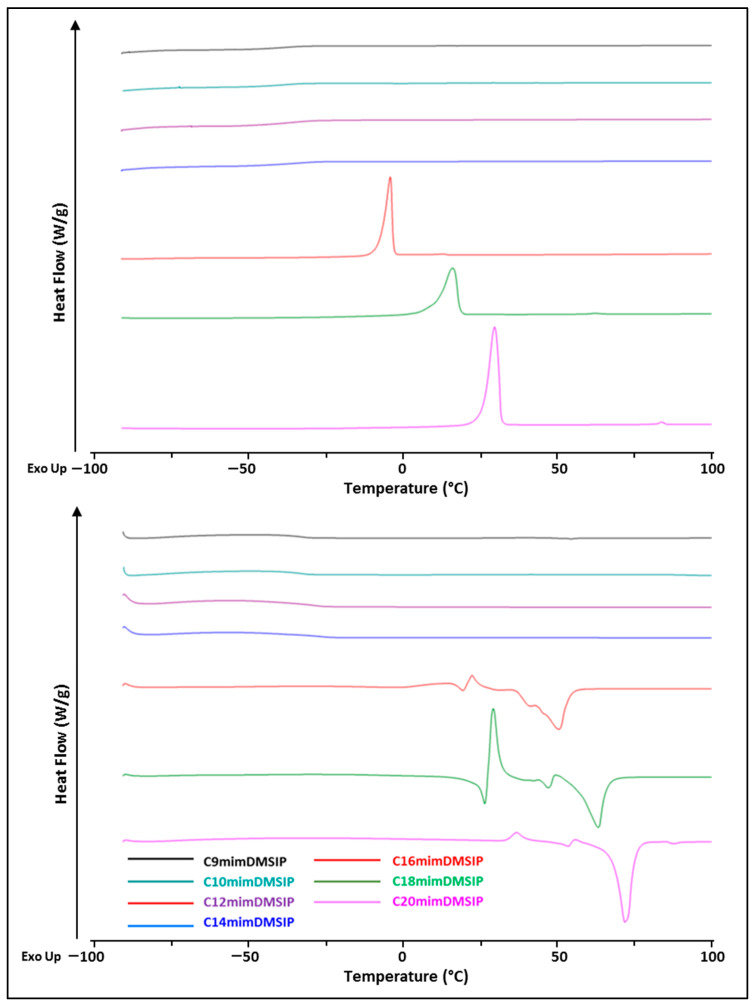
DSC curves of the series C*n*mimDMSIP at 10 °C/min cooling and heating runs. (**Up**) cooling cycle; (**Down**) second heating cycle. Curves are displaced for clarity. The legend refers to both graphics.

The ILs of the second group display only glass transition temperatures, indicating the formation of amorphous glass with no melting or freezing points. The ILs exhibiting this behaviour in the study are C9mimBF_4_ (T_g_ = −75 °C), C9mimDMSIP (T_g_ = −33 °C), C10mimDMSIP (T_g_ = −33 °C), C12mimDMSIP (T_g_ = −32 °C) and C14mimDMSIP (T_g_ = −32 °C) (Figure 2 and Figure 3).

The ILs belonging to the third group do not crystallise during the cooling cycle. Still, they display glass transition, cold crystallisation, and melting peaks upon heating, showing a polymorphic behaviour similar to that of polymers and other amorphous materials [70,71]. ILs such as C12mimBF_4_, C14mimBF_4_, C16mimDMSIP, C18mimDMSIP, and C20mimDMSIP reveal polymorphism upon a second heating scan, with T_cc_ values of 1, 25, 22, 28, and 35 °C and T_m_ values of 31, 39, 49, 62, and 70 °C, respectively. However, during this scan, T_g_ was not observed. Moreover, differently from the literature, the thermal behaviour described for ILs of the third group also shows a freezing transition upon cooling, displaying T_c_ values of 0, 24, −4, 16, and 29 °C, respectively.

As previously noted [70], the grouping of ILs based on thermal behaviour does not fully capture the various types of polymorphisms or the different behaviours that ILs can exhibit at different cooling/heating rates. Similar thermal behaviour can be observed among ILs composed of different cations with the same or different anions, such as C10mimBF_4_, C12mimBF_4_, C14mimBF_4_, C16mimDMSIP, C18mimDMSIP, and C20mimDMSIP.

Our findings align with those of a prior study reporting the synthesis and characterisation of a series of 1-alkyl-3-methylimidazolium tetrafluoroborates ionic liquids and ionic liquids crystals [72]. The authors found that, at RT, the ILs bearing short alkyl chains (*n* = 2–10) were isotropic, showing a diverse liquid range. Conversely, the ILs with longer alkyl chains were low-melting mesomorphic crystalline solids, revealing an enantiotropic smectic mesophase. The thermal range of these mesophases increased with the lengthening of the alkyl chains. In a recent study of a C*n*mimBF_4_ series (*n* = 0–12) [73], the authors used Electron Paramagnetic Resonance (EPR) spectroscopy to analyse the ILs. They found nanocage formation and structural anomalies in the ILs.

Considering the T_g_ values of both IL series, we found a value for C9mimBF_4_ (−75 °C) which is close to that (from −83 °C to −77 °C) reported in the literature [72,73], whereas for the series constituted by the anion DMSIP, there are no literature data to compare with our results, with it being synthetised for the first time.

The series C*n*mimBF_4_ (*n* = 9–18) showed increasing values of T_m_ (−4 to 59 °C) and T_c_ (−33 to 53 °C) as the chain length increases, comparable to those from the literature [72,73]. In particular, C14mimBF_4_ displayed T_c_ and T_m_ values of 24 and 39 °C, close to those reported previously [72]. As expected, DSC analysis of C20mimBF_4_ showed the highest T_c_ (64 °C) and T_m_ (70 °C) values.

The series of ILs containing the DMSIP as the anion showed a different behaviour, as described above, for their grouping. The ILs with alkyl chain lengths of *n* = 9–14 exhibited similar T_g_ values, ranging from −33 to −32 °C. They tended to be supercool, forming viscous liquids and glasses without crystallisation and melting peaks on cooling and heating cycles. The ILs with *n* = 16–20 showed a polymorphic behaviour, with no T_g_ observed under the experimental conditions adopted. These ILs had thermal transitions relative to T_c_ upon cooling, and the T_cc_ and T_m_ peaks during the second heating run. All thermal transitions showed a shift of T_c_, T_cc_, and T_m_ at higher temperatures with the increasing length of the alkyl chain in the cations. Weakly defined endothermic transitions of overlapped peaks of different crystals or solid-solid transition melting with their quick recrystallisation preceded true T_m_.

PVC/IL blends were produced using neat PVC plasticised with TOTM to elude the exposure of phthalates, which can cause harm to the environment and human health. DSC analysis of the neat PVC showed a glass transition temperature (T_g_) of 63 °C during the first heating cycle, whereas T_g_ was not observed during the second cycle (Figure S4). Likewise, the PVC blends loaded with the different concentrations of the ILs chosen displayed visible T_g_ of the matrix only on the first heating cycle. Moreover, the PVC blends loaded with 5% of C*n*mimBF_4_ and C*n*mimDMSIP also showed T_m_ of the embedded ILs during the first heating cycle (Figures S5 and S6); nevertheless, these transitions were not depicted during the second heating cycle of the PVC blends loaded with the C*n*mimDMSIP, probably hidden by the TOTM-induced plasticisation, as previously observed [17]. In the case of PVC blends containing ILs from the C*n*mimBF_4_ series, T_m_ of ILs was also observed during the second heating (Figure S5), suggesting a minor plasticising effect and compatibility with the PVC matrix.

Thermogravimetric analyses were performed to evaluate the thermal stability of ILs and PVC/IL blends. Before testing, the samples were subjected to drying cycles in a vacuum stove for 24 h at 50–60 °C, according to the thermal features of ILs, mainly considering their melting points. The ILs C9mimBF_4_, C10mimBF_4_, C9mimDMSIP, and C10mimDMSIP, which are liquid at RT, were left to dry at 30 °C for 4–5 h. The thermogravimetric analyses performed from 30 °C to 800 °C resulted in similar values to those obtained from 40 to 600 °C, with the only slight difference due to a decrease in the residue (up to 2 wt%). Considering this, the 40–600 °C degradation temperature range was adopted for the subsequent TGA analyses. These short-term analyses were conducted at a heating rate of 10 °C/min to obtain results comparable with literature data [72,74]. Furthermore, platinum pans and nitrogen as purge gas were used, and the temperature at which 5% weight loss occurred was defined as T_onset_, aiming to avoid the decomposition differences due to pan and gas types, as well as the uncertainty from the manual determination of the T_onset_ tangent point, respectively [71,75,76,77]. Many features can influence the thermal stability of ILs, mainly the coordinating nature of the anion. Indeed, the highly coordinating halide anions (Cl, Br) display a thermal stability lower than that of poor coordinating anions (PF_6_) [78,79]. The type of cation core influences the thermal stability of ILs, with a decreasing temperature resistance in the following order: pyrrolidinium, imidazolium, pyridinium, and non-cyclic tetra-alkyl ammonium. The increasing alkyl chain length of the imidazolium cation and branching of the alkyl substituents induce a reduction in thermal stability. Furthermore, a decrease in thermal stability is determined by the presence of alkoxylated side chains in methylimidazolium, piperidinium, and pyrrolidinium cations [17,77].

**Figure 4:**
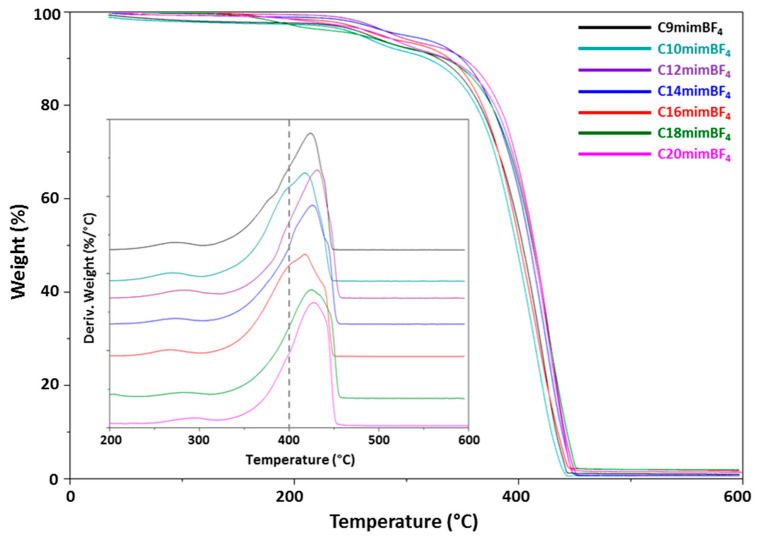
TGA curves of the synthetised ILs containing BF_4_ as the counter ion. The DTG curves of the inset are displaced for clarity. The legend refers to both graphics.

**Figure 5:**
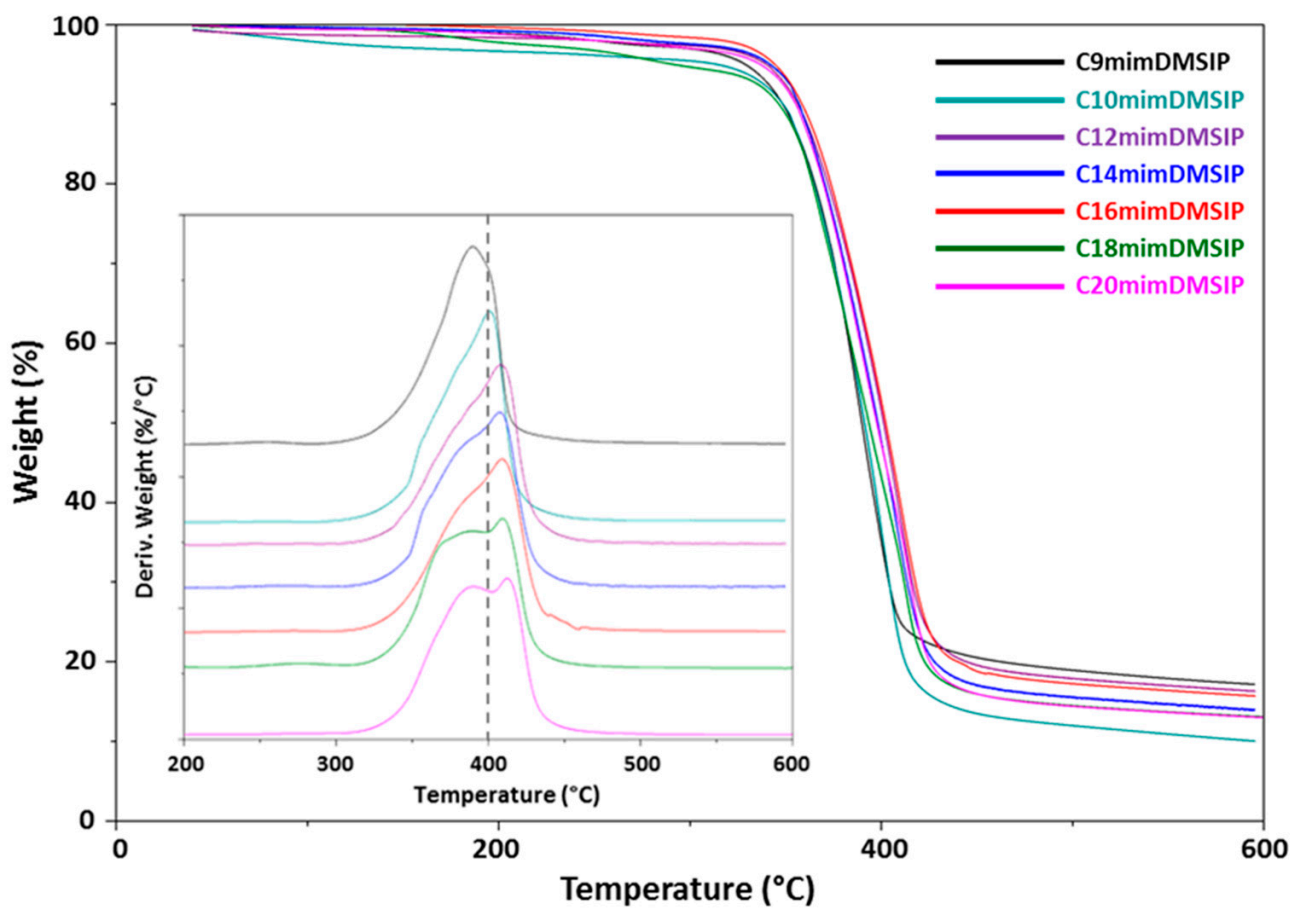
TGA curves of the synthetised ILs, comprising DMSIP as the counter ion. The DTG curves of the inset are displaced for clarity. The legend refers to both graphics.

TGA and DTG curves of the studied ILs are reported in Figure 4 and Figure 5. The ILs belonging to the BF_4_ series (Figure 4) showed 5% weight loss and maximum degradation temperature (T_d_) above 250 and 400 °C, respectively, which are in agreement with the literature data [72,73].

The ILs from the DMSIP series (Figure 5) exhibited 5% weight loss above 300 °C and were stable up to ca. 410 °C. Degradation of ILs of both series happened in a single step, recording Td values of the ILs belonging to the BF_4_ series between 417.2 °C (C10mimBF_4_) and 427.9 °C (C20mimBF_4_), whereas those of the DMSIP series were slightly lower, between 389.7 °C (C9mimDMSIP) and 412.2 °C (C20mimDMSIP) (Figure 4 and Figure 5).

These results demonstrated the thermal stability of both series of the synthetised ILs. As a result, the most promising ILs, selected based on their antibacterial properties, were loaded into PVC.

As the industrial production of end products usually involves processing methodologies during which undesirable degradation processes could affect materials’ performance, the thermal stability of neat PVC and PVC/IL film blends was analysed. As detailed for TGA analyses of pure ILs, the stability of the PVC/IL blends was determined at heating runs of 10 °C/min from 40 to 600 °C under a nitrogen atmosphere using a platinum pan. These things considered, the temperature at 5% weight loss was considered as T_onset_.

Table 2 reports TG and DTG data of neat PVC and PVC/IL blends. The thermal degradation of neat PVC happens in two steps. The first step, caused by the loss of hydrochloric acid, and the second one, relative to the degradation of newly formed polyenic chains, occur in the ranges of 200–300 °C and 360–450 °C, respectively [80].

As previously discussed, adding ILs to both series did not affect the appearance and consistency of the PVC film blends, which resulted in softness even at the highest concentrations of filled ILs (Figure S3). Only the film loaded with the IL C16mimBF_4_ showed a slight opacity (Figure S3F).

All PVC blends did not show remarkable differences from neat PVC at temperatures below 200 °C. Nevertheless, at temperatures above 200 °C, a decrease in the onset temperature of all PVC/IL blends with an increase in the IL concentration in the PVC matrix was observed. Similar behaviour was registered during the first step of degradation with a lowering of T_d1_, whereas during the second step of thermal degradation (T_d2_), slight differences (up to 5 °C) between neat PVC and PVC/IL blends occurred.

The T_onset_ and T_d1_ decrease was dependent on both IL concentration and structure. Considering T_onset_, the highest lowering was observed with the highest concentration of ILs. Moreover, the blends containing the C12mim cation of both series displayed the highest difference from neat PVC, as shown in Table 2. Indeed, the decrease in T_onset_ was in the temperature range of 40–49 °C for the PVC/IL blends containing C16mimBF_4_ and C12mimBF_4_, respectively, and slightly less (32–44 °C temperature range) for the films loaded with the ILs C16mimDMSIP and C12mimDMSIP. Similarly, the T_d1_ differences ranged from 75 °C (C16mimBF_4_) to 86 °C (C12mimBF_4_) and from 62 °C (C16mimDMSIP) to 77 °C (C12mimDMSIP) between the blends and neat PVC. These data indicate that both the anion and cation nature contribute to the thermal stability of PVC blends. Indeed, even if no difference higher than 13 °C was observed between the two blends series, the ILs containing the DMSIP anion seem to make PVC blends slightly more stable than those bearing the BF_4_ anion. This is probably due to their different coordinating nature and affinity vs. the PVC matrix, as previously observed for another IL [17]. The thermal stability induced by the cation depends on chain length, increasing as the alkyl chain length increases. The lower thermal stability of PVC/IL blends with respect to neat PVC is probably due to IL contribution to the aforementioned autocatalytic degradation of PVC, anticipating or delaying the dehydrochlorination of chlorine atoms from PVC chains.

Similar lowering or enhancement of maximum degradation temperature was previously reported for other polymers such as Pebax Rnew, SEBS, PMMA, PBT, PC, and PET [15,19,43,48,49,50].

In general, the residue of PVC blends of both series, observed at 600 °C, showed a slight decrease (1–4%) with respect to the value observed for neat PVC (9%). In particular, lower values were found for the blends containing the C*n*mimBF_4_, whereas they were similar to those of neat PVC for the PVC/C16mimDMSIP at 0.5–1 wt%, with a slight increase in the highest concentration (5 wt%) of the IL. As previously observed [17], this may be due to the IL’s participation in the polyenic chains’ crosslinked complexes, which do not degrade up to 600 °C.

### 3.3. Morphological Investigations: Scanning Electron Microscopy (SEM)

SEM analysis allows for exploring the surface structure of polymers together with the dispersion, orientation, shape, and size of fillers loaded into them. The loading of ILs into the PVC matrix determined a polymer surface modification mainly depending on their concentration and structure.

**Figure 6:**
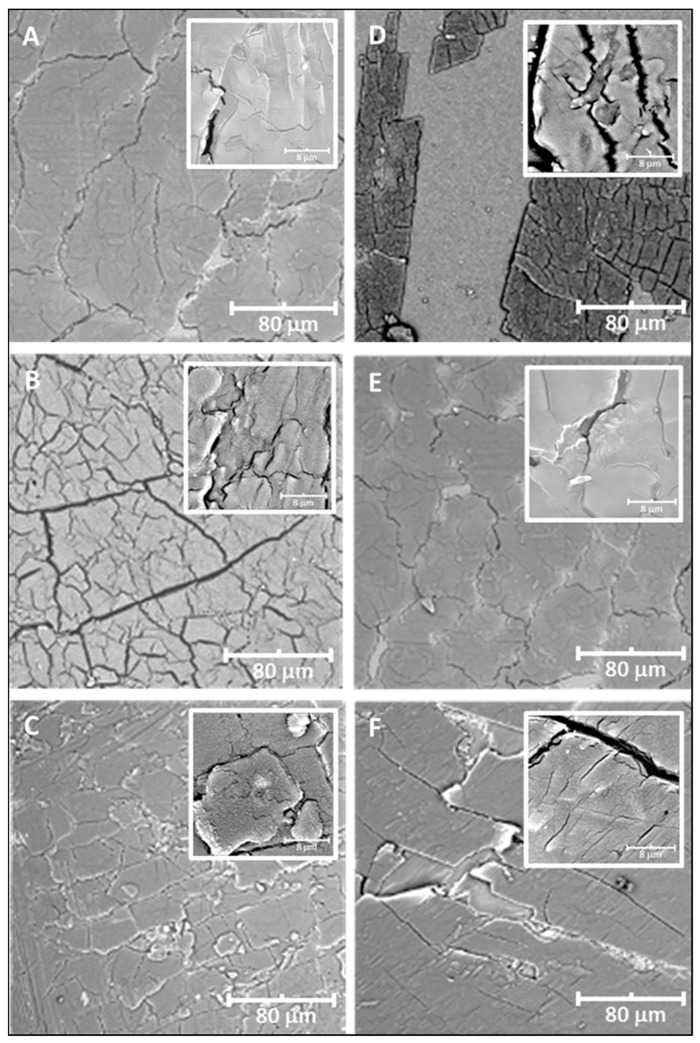
SEM images (magnification 1000×) of PVC blend films containing 5 wt% of (**A**) C12mimBF_4_, (**B**) C14mimBF_4_, (**C**) C16mimBF_4_, (**D**) C12mimDMSIP, (**E**) C14mimDMSIP, (**F**) C16mimDMSIP. Inset magnification 10,000×.

Neat PVC displayed a smooth surface without precise characteristics (Figure S5), whereas its IL blends showed morphological modifications due to irregularly distributed aggregates on the polymer surface. These aggregates were of variable shape and size depending on their concentration and were poorly dispersed in irregular small spots in the PVC blends containing 0.5 wt% (Figure S8) of all ILs and more abundant in those (Figure S9) with a higher concentration of ILs (1 wt%). The blends containing 5 wt% ILs (Figure 6) exhibited significant modifications of polymer morphology, with noticeable dense aggregations of ILs distributed prominently across the polymer surface. The addition at this concentration of both types of ILs, characterised by varying lengths of alkyl chains in the cations and different counterions, resulted in the creation of irregular structures densely scattered on the polymer surface, except for the PVC/5 wt% C12mimDMSIP. Indeed, this blend exhibited a less compact morphology of the irregular like-island structures, which did not uniformly cover the polymer surface (Figure 6D). Furthermore, several cracks were present between the structures surrounding the film surfaces, more evident in PVC/5 wt% C14mimBF_4_ and others with irregular structures located on those distributed compactly and uniformly over the entire surface (PVC/5 wt% C16mimBF_4_ and PVC/5 wt% C16mimDMSIP).

PVC/5 wt% C16mimDMSIP also displayed an oriented IL deposition, particularly evident in the inset of Figure 6F, as previously observed [17]. Incorporating the ILs C_16_mimCl and C_16_mimMeS into a PLA matrix consisting of cations with the same alkyl length but differing anions from DMSIP led to distinct surfaces (spheres or lines). These morphologies were dependent on the concentration of ILs and their interaction with PLA [51].

### 3.4. Contact Angle Measurements

**Figure 7:**
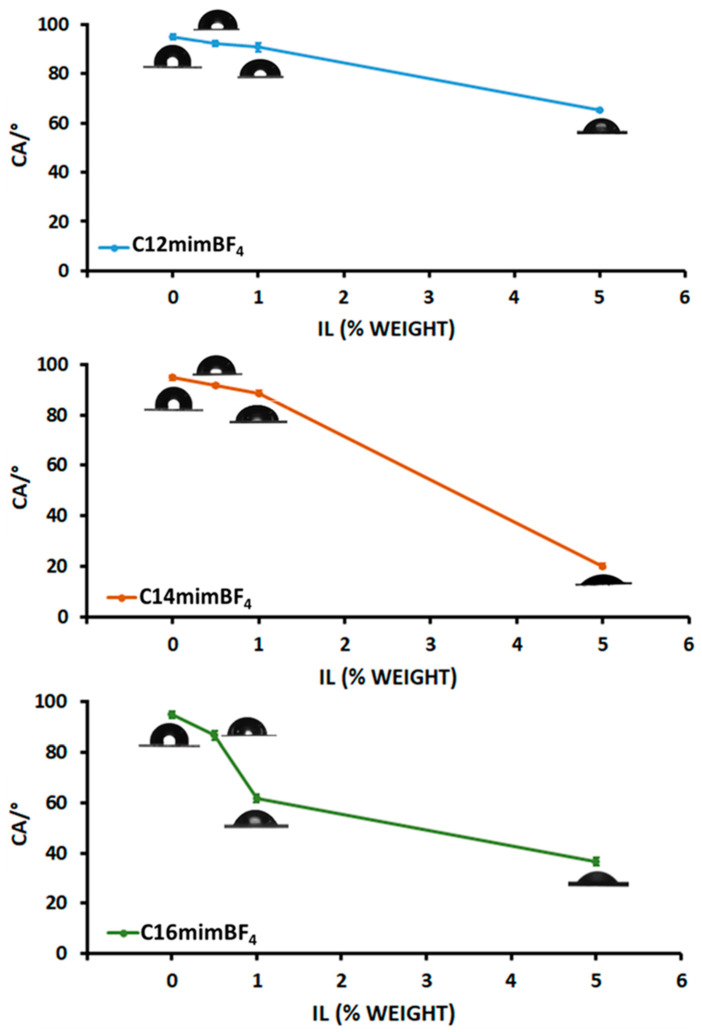
Water contact angle of PVC/C*n*mimBF_4_ blends as a function of IL weight percent.

**Figure 8:**
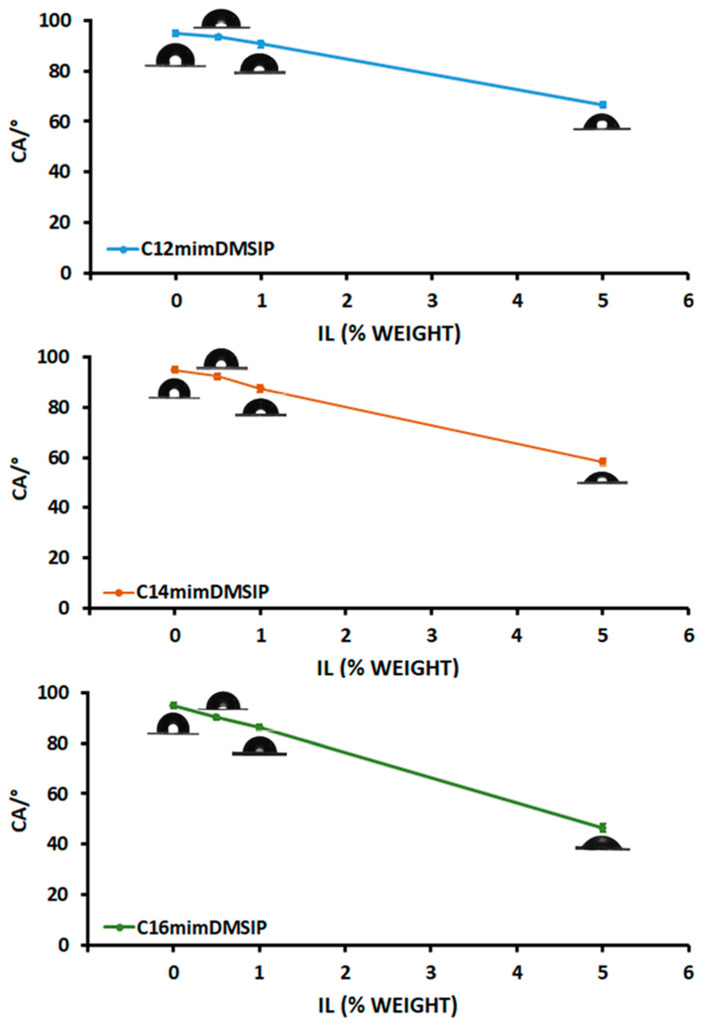
Water contact angle of PVC/CnmimDMSIP blends as a function of IL weight percent.

Measurements of the water contact angle (WCA) were carried out to determine the wettability of neat PVC and PVC/IL films. The influence of different concentrations (0.5, 1, and 5 wt%) of ILs on PVC blends was also explored. Neat PVC (Figure 7 and Figure 8), being a hydrophobic material, showed a WCA value of 94°, whereas all the PVC blend films loaded with both series of ILs displayed a decreasing trend of WCA values, mainly dependent on IL content.

Polymer wettability is influenced by the additives’ structure, chemical composition, and surface texture of the polymers (size and shape of particles, roughness). The increase in surface roughness decreases the CA values of hydrophilic materials and increases those of hydrophobic materials [81].

In the case of ILs, elongating the alkyl chain length of the cation increases hydrophobicity. Conversely, including ether and ester groups in the alkyl chain decreases the octanol–water partition coefficient, reducing the ILs’ lipophilicity [78,82]. Moreover, anions’ coordination ability (H-bonding capability) influences ILs hydrophobicity/hydrophilicity [78].

The PVC blends examined in this study comprised various concentrations of ILs. The findings revealed that increasing amounts of ILs from both series reduced the polymer’s hydrophobicity, particularly at the highest IL content (5 wt%). This indicates a dose-dependent behaviour, with a decisive contribution of the IL concentration on the wettability of the blends. Considering the BF_4_ series, a decrease in CA values from 94° for neat PVC to 65°, 20° and 37° for PVC blends containing 5 wt% of C12mimBF_4_, C14mimBF_4_ and C16mimBF_4_, respectively, was observed. In the case of the DMSIP series, CA values of 66°, 58°, and 47° were found for the PVC blends loaded with 5 wt% of C12mimDMSIP, C14mimDMSIP, and C16mimDMSIP, indicating not only a dose-dependent behaviour of surface morphology, but also a dependence on the alkyl chain length of ILs. While loading the highest concentration of ILs did result in surface roughness of the PVC blends, as evidenced by SEM analysis, the incorporation of ILs into the PVC matrix brought about a distinct reduction in the contact angle compared to that of the neat polymer. This indicates that the nature of the ILs likely has a more significant impact than that of surface roughness, as previously noted [15,17]. Although the highest concentration of ILs induces roughness on the PVC surface, varying ILs at the same concentration result in different CA values. This observation suggests a stronger hydrophilic effect of BF_4_ anion than that of DMSIP, especially notable with C14mimBF_4_ and C16mimBF_4_ at a concentration of 5 wt%.

### 3.5. IL Release from the PVC/IL Blends

**Figure 9:**
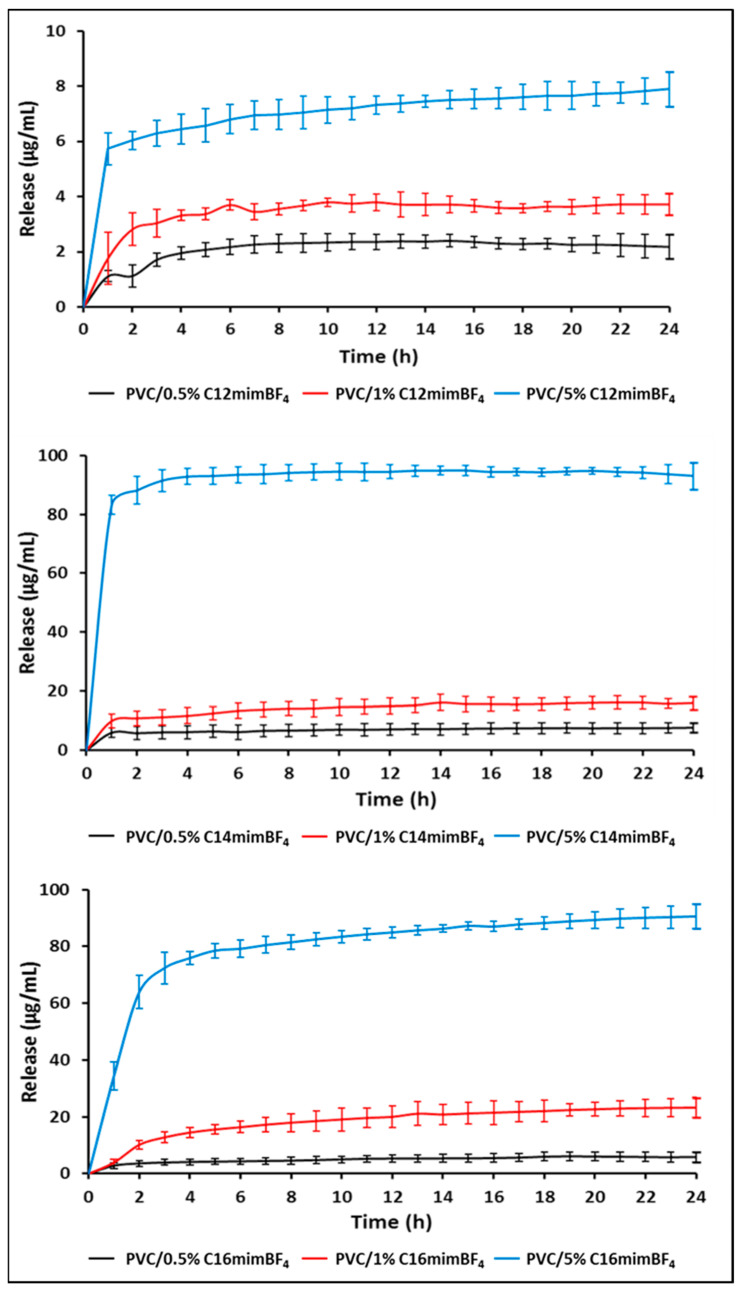
IL release from PVC/C*n*mimBF_4_ blends. Points are the mean of three independent replicates with *n* = 24 each. Bars represent ± SD.

**Figure 10:**
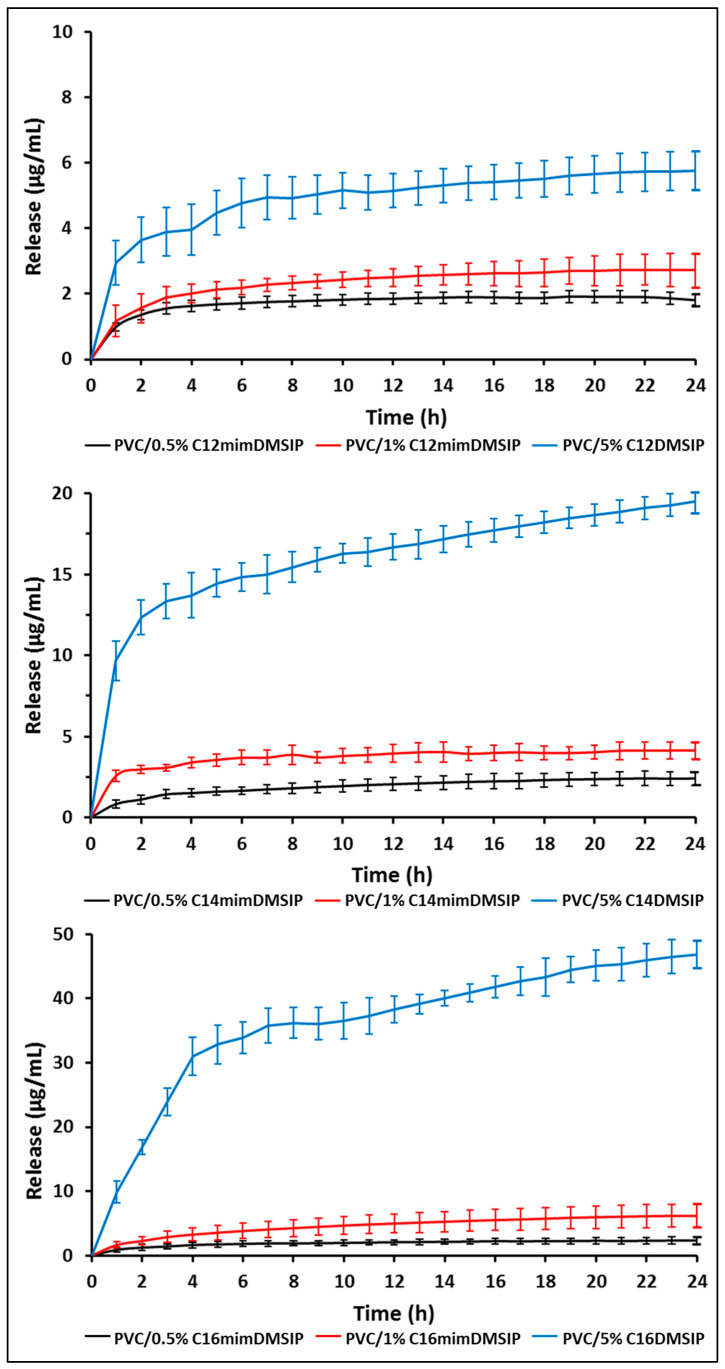
IL release from PVC/C*n*mimDMSIP blends. Points are the mean of three independent replicates with *n* = 24 each. Bars represent ± SD.

The release of ILs from the PVC blend films loaded with ILs at concentrations 0.5, 1, and 5 wt% was investigated in a biomimetic physiological solution such as PBS (10 mM, pH 7.4) at 210 nm using UV–vis spectrophotometry. The IL release profiles (µg/mL) are reported in Figure 9 and Figure 10.

Results underlined a different IL release from the PVC blends depending on cation chain length, type of anion, and time. Considering the chain length, for the series with BF_4_ anion, the release followed the trend C12 < C14 ≅ C16 (Figure 9). The release from the PVC blends loaded with 5 wt% ILs from the BF_4_ series was as follows: 7.89, 83.2, and 34.4 µg/mL (1 h), as well as 7.89, 93.1, and 90.6 µg/mL (24 h) for C12, C14, and C16, respectively. For the IL series with the DMSIP anion, the similar release trend C12 < C14 < C16 was observed, with release values from the PVC blends loaded with 5 wt% ILs of 2.95, 9.64, and 9.85 µg/mL (1 h), as well as 5.76, 19.48, and 46.83 µg/mL (24 h) for C12, C14 and C16, respectively. The lower release observed for C12mim may be attributed to its better compatibility with the polymeric matrix than the C14mim and C16mim chains. Regarding the type of anion, the release data (Figure 9 and Figure 10) revealed a more rapid and pronounced release from the blends loaded with ILs containing BF_4_ compared to that of DMSIP across all concentrations added to PVC. This behaviour is consistent with the better compatibility of the C*n*mimDMSIP ILs with the PVC matrix, as discussed earlier [15,17,19].

In Table S3, the percentages of release from the PVC blends of the two series are reported. After 24 h, at different concentrations, the PVC/C12mimBF_4_ blends showed a release in the range of 2.6–5.9%, whereas PVC/C14mimBF_4_ and PVC/C16mimBF_4_ blends displayed a higher release in the range of 23.8–30.4% and 18.5–29.6%, respectively. In the case of PVC blends loaded with the IL series containing the DMSIP anion, the release for PVC/C12mimDMSIP and PVC/C14mimDMSIP was in the range of 1.8–4.2% and around 6–7%, respectively. A higher release in the 6.5–16.3% range was recorded for the PVC/C16mimDMSIP blends.

The differences in the release percentages are due to various factors. In addition to the above-discussed influence of the alkyl chain length and type of anion, the interaction between the ILs with the polymer matrix, as well as the diffusion properties of the components, play an important role.

These findings underscore the complex interplay between the chemical composition of the ILs, their interaction with the PVC matrix, and the resulting release kinetics. Such insights are crucial for optimizing the performance of PVC-based systems in various applications, particularly in controlled release scenarios.

### 3.6. Antibacterial Activity

#### 3.6.1. Antibacterial Activity of Neat ILs

The proliferation of bacterial infections and the relentless spread of antibiotic-resistant bacteria pose a serious challenge to human health and safety. Additionally, the increasing incidence of diseases arising from contaminations in everyday products contributes to morbidity and mortality. Several research efforts have been undertaken to find valuable alternatives to conventional preservatives and antibiotics. Over the past few decades, many types of ILs have been extensively studied for their interesting antimicrobial properties. In this study, two series of ILs were developed and characterised with varying alkyl chain lengths of the cations and different types of anions to evaluate their antibacterial activity against three bacterial species (*Listeria monocytogenes*, *Escherichia coli*, *Pseudomonas fluorescens*) using the minimum inhibitory concentration (MIC) and the minimum bactericidal concentration (MBC) determined by the broth dilution method (Table 3).

The results revealed a different antimicrobial behaviour of both series of ILs, primarily dependent on cation chain length and secondarily on the type of anion. Furthermore, their antibacterial efficacy varied based on the specific bacterial strain analysed and, in particular, on the structural difference between Gram-negative and Gram-positive cells appearing in some cases to be more effective against Gram-positive ones. Structurally, Gram-positive bacteria comprise a much thicker porous cell wall made up of peptidoglycan layers interlinked with each other by a negatively charged teichoic acid, which is responsible for its porosity. On the contrary, the Gram-negative bacterial cell wall comprises two layers: the outer one is negatively charged and made up of lipopolysaccharides, and the inner one is relatively thin and made up of peptidoglycan. Therefore, it is hypothesised that the hydrophobic part of IL is more likely to be inserted into the porous peptidoglycan, disrupting the cell membrane and causing cell death. In the case of Gram-negative bacteria, the lower permeability of the outer membrane protects the cell from the entry of antibacterial compounds. Ionic liquids are ascribed to the ability to perturb the biochemical gradients between the cell cytoplasm and the external environment, resulting in the penetration of extracellular materials into the cytoplasm or diffusion of the intracellular contents out of the cell [83].

Considering the chain length of cations of the series with BF_4_ anion, the activity against the growth of *L. monocytogenes*, expressed in µM, was in the order C18mim = C9mim < C10mim < C16mim < C12mim < C14mim, whereas C20mim did not display antibacterial activity at the maximum concentration used (1000 µM, 450.45 µg/mL).

The activity against the growth of *E. coli* increases in the order C9mim < C10mim < C18mim < C12mim < C16mim < C14mim. Again, C20mim did not show antibacterial activity at 1000 µM (450.45 µg/mL). The same IL series displayed an antibacterial activity vs. *P. fluorescens* lower than that showed against the other two strains, following the increasing trend C10mim = C16mim < C12mim < C14mim. The ILs with the C9, C18, and C20 cation chain length did not exhibit growth inhibition and/or cell death of this strain at their maximum concentration (1000 µM).

Regarding the series with the DMSIP anion, the antibacterial activity against the same strains was slightly different, showing the trends C18mim < C10mim < C14mim = C16mim < C12mim vs. *L. monocytogenes* and C9mim = C18mim < C10mim < C16mim < C12mim < C14mim against *E. coli*, with no inhibition of the IL bearing the C20 chain at the maximum concentration used (1000 µM, 636.88 µg/mL). The same behaviour of the series comprising the BF_4_ anion was observed in the case of *P. fluorescens* growth inhibition, with no inhibition by the ILs containing C9, C18, and C20 alkyl chains. The antimicrobial activity against *P. fluorescens* resulted mainly from the alkyl chain length of the cations, regardless of the type of anion.

On the whole, it is possible to note that the ILs most active against L. monocytogenes do not always give fair results, even against the tested Gram-negative bacteria. Specific studies should be conducted to evaluate better the mechanism of action of the ILs under study towards Gram-positive and Gram-negative bacterial strains.

The ILs with the cations containing C12, C14, and C16 alkyl chains showed the best antibacterial activity against all tested bacterial strains. These findings are in accordance with those of a previous study regarding the antimicrobial activity of two series of 1-alkyl-3-methylimidazolium chlorides and bromides [5]. Nevertheless, some differences were detected considering the type of anion. C14mimBF_4_ showed the highest antibacterial activity against all strains under study (Table 3). In detail, the series containing the BF_4_ anion showed MIC values against *L. monocytogenes* ranging from 500 µM for C9mimBF_4_ and C18mimBF_4_ (148.08 and 211.2 µg/mL, respectively) to 12.5 µM (4.58 µg/mL) for C14mimBF_4._ In the case of *E. coli*, the MIC values were in the range of 1000–12.5 µM (296.16–4.58 µg/mL) for ILs C9mimBF_4_ and C14mimBF_4_, respectively. Antibacterial activity against *P. fluorescens* was exerted only by the cations having C10, C12, C14, and C16 alkyl chains, with MIC values ranging from 125 µM (C10mim and C16mim) to 25 µM (C14mim).

The DMSIP anion series showed MIC and MBC values lower than those of the parent BF_4_ series. This indicates that antibacterial activity also depends on the nature of the anion. Indeed, the anion’s poor coordinating nature and less steric hindrance could increase ILs’ antimicrobial effect [37].

Our findings are consistent with those of the existing literature data. Several studies reported that the antimicrobial activity of ILs arises from electrostatic interactions between the ILs cations and bacteria’s negatively charged cell walls. Specifically, the hydrophobic alkyl chains of IL cations attach to the phospholipid bilayer, disrupting the hydrophobic bacterial cell membrane and ultimately leading to bacterial cell death [36,37,39,84]. It has been demonstrated that the antimicrobial activity of ILs increases with the elongation of the alkyl side chains. ILs with long alkyl chains (10 to 14 carbon atoms) exhibit high antimicrobial activity [5,37,39,85]. ILs display maximum activity with 14 carbon atoms in the alkyl chain, whereas the further elongation of the alkyl chain does not lead to an increase in IL toxicity due to the “cut-off” effect [36,37,86]. The antibacterial mechanism exerted by ionic liquids is ascribed to four phases: I) adsorption to the cell membranes; II) electrostatic interaction between cationic moieties of the ionic liquids and the functional groups of the phospholipid bilayer; III) penetration, disorganisation, and disintegration of the phospholipid bilayer; IV) cell wall destruction and lysis. The penetration of bacterial membranes allows ionic liquids to disrupt membrane potential viscoelasticity, fluidity, and the arrangement of phospholipids, allowing them to enter the cytosol. This alteration in membrane fluidity leads to changes in the stability and diffusion rate of the membrane proteins, subsequently affecting molecule transportation, migration, adhesion, membrane permeability, and formation of pores [36,38,59,83,84,87,88,89].

It was observed that the highest antibacterial activity was exhibited by ILs from both series, with cations possessing C12, C14, and C16 alkyl chains. Among these, C12mim and C16mim ILs in both series displayed varying MIC and MBC values depending on the sensitivity of the bacterial strain. Notably, C14mimBF_4_ exhibited the lowest values for all examined strains. Specifically, the increased activity was observed against *L. monocytogenes*, consistent with the reported higher sensitivity of Gram-positive bacteria [5,15,17,19,85,86] compared to that of Gram-negative ones. Generally, the obtained MIC values of the ILs align with the antimicrobial activity of other imidazolium-based ILs [84,85,89]. Nevertheless, differences from the literature data may be ascribed to variations in the methodologies and composition of media used to perform the antimicrobial tests, bacteria initial concentration, time exposure, and the different sensitivity of the strains tested [5,39,85,90,91].

#### 3.6.2. Antibacterial Activity of PVC/IL Blend Films

Circular samples of PVC/IL blend films (4 cm^2^ surface) were used to assess the antimicrobial activity of the investigated blends. The agar diffusion method was employed to ascertain the potential inhibition of bacteria growth induced by the PVC/IL blend films. Table 4 presents the inhibition zones induced by PVC samples loaded with increasing concentrations of both series of ILs, along with neat PVC for comparison, on NA spread-inoculated with three bacterial strains (*L. monocytogenes*, *E. coli*, *P. fluorescens*), at a concentration of 10^6^ CFU/mL. The inhibition zone values observed for both PVC/IL blend series (Table 4) are consistent with the data obtained from SEM, contact angle, and release analyses. Specifically, PVC blends containing 5% ILs showed the highest values due to the accumulation of IL on the film surfaces (Figure 6, Figure 7 and Figure 8) and the subsequent fast release, particularly for the BF_4_ series (Figure 9 and Figure 10). This suggests that the 5% IL concentration may be excessive for homogenous incorporation into the PVC matrix.

**Figure 11:**
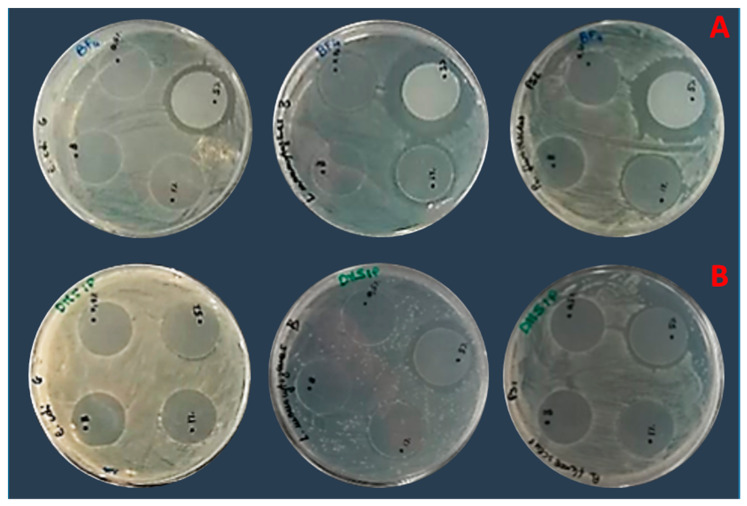
Images of inhibition halos induced by PVC/C14mimBF_4_ (**A**) and PVC/C14mimDMSIP (**B**) against *Listeria monocytogenes*, *Escherichia coli*, *Pseudomonas fluorescens* growth spread on NA plates at a concentration of 10^6^ CFU/mL.

Considering the antimicrobial activity of PVC blends loaded with the C*n*mimBF_4_ series, the lower compatibility and reduced steric hindrance of the BF_4_ anion contribute to increased IL release and, consequently, the antimicrobial effect of their respective PVC blends. The PVC/C14mimBF_4_ blend exhibited the most effective antimicrobial activity against the growth of the three strains analysed, as illustrated in Figure 11A. Conversely, the series containing the DMSIP anion demonstrated greater compatibility with the PVC matrix, thus impeding the release of ILs at lower concentrations. Alternatively, the 5% concentration, as observed in the BF_4_ anion series, was too high for stable integration into the matrix. Consequently, it migrated to the polymer surface and was released, producing good antibacterial activity against all tested strains (Figure 11B).

### 3.7. Biocompatibility/Toxicity of ILs

In searching for novel antitumor agents capable of avoiding the development of drug resistance and overcoming limitations due to the lack of tumour specificity of many drugs, numerous ILs were investigated to determine their antitumor activity [53,54,56,57,58,59,92,93]. Nevertheless, since many ionic liquids are water-soluble, humans and animals may be exposed to them both during and after as chemical waste. Consequently, studies on IL toxicity are crucial and indispensable, as they enable us to predict toxicity among ILs and conduct accurate risk assessments. Moreover, the biocompatibility of a material is the primary factor determining its suitability for biomedical applications [51,52]. In general, in vitro cell attachment and proliferation on material surfaces are commonly employed as crucial preliminary assessments of cell biocompatibility.

The impact of ILs on healthy and cancer cells can vary significantly, primarily determined by their distinct chemical compositions and mechanism of action. It is essential to recognise that many ionic liquids exhibit cytotoxic effects, indicating they can harm healthy and cancerous cells [88,93]. However, the degree of this cytotoxicity varies widely. Certain ionic liquids have demonstrated a higher toxicity towards cancer cells while being less harmful to healthy cells. This specificity, also influenced by IC_50_, is often linked to variations in membrane composition and metabolic activity between normal and cancer cells. This behaviour may be attributed to various factors, including the physicochemical properties of ILs and different cell characteristics. Notably, cancer cells often possess more delicate membranes than those of healthy cells, making them more vulnerable to such damage. Nonetheless, this effect depends on the alkyl chain length and the anion groups. Several studies suggest that when combining head groups such as imidazolium, ammonium, phosphonium, and pyridinium with alkyl side chains ranging from C1 to C18, the toxic effects increase with longer alkyl side chains. This results in decreased cell viability, a pattern supported by numerous investigations with various human cell lines. For example, in comparing C4mim (1-butyl-3-methylimidazolium) and C8mim (1-octyl-3-methylimidazolium), regardless of the substituent anion (BF_4_ or PF_6_), C8mim was found to reduce cell viability more than C4mim against the human colon adenocarcinoma cell line (HT-29) [50]. ILs can cause cell death by necrosis or apoptosis by influencing cellular metabolism or disrupting cell membranes by breaching the membrane [61]. Some studies reported that ILs with *n* = 6 and 8 make the lipid membrane more permeable in comparison to *n* = 4 using methylimidazolium cation [Cnmim]^+^ (*n* = 4, 6 and 8) [84]. These ILs completely demolish the lipid bilayer at a dose of 200 mM. The identical cation [bmim]^+^ but distinct anions [BF_4_]^−^ and [PF_6_]^−^ were used in two separate RTILs obtained by Benedetto et al. [87]. The research has shown that [bmim]^+^ is successfully incorporated into the lipid bilayer, showing a systematic weakening of the bilayer. Furthermore, because different types of mammalian cells share many characteristics, researching how the cells react to ILs can reveal general information on imidazolium IL cytotoxicity.

Here, the antitumor activity and cytotoxicity of the synthetised imidazolium-based ionic liquids have been determined for breast cancer cells (MCF-7) and healthy human fibroblast (HDF) cells. We evaluated the ILs’ effect on healthy and cancer cells by comparing their IC_50_ values, as shown in Table 5. These IC_50_ values were calculated after 24 h of cell incubation in solutions containing various concentrations of the IL series C*n*mimBF_4_ and C*n*mimDMSIP, indicating the concentration at which these ILs exhibit 50% inhibition of cell growth or viability after 24 h of exposure. Table 5 reports the IC_50_ values in µg/mL and µM to make their comparison with the literature data easy.

Upon comparing the IC_50_ results for MCF-7 and HDF cells, expressed in both µg/mL and µM, a notable trend emerges. For several IL groups, IC_50_ associated with MCF-7 is lower than that associated with HDF cells, indicating a relatively higher sensitivity of cancer cells to these compounds than healthy cells. This discrepancy suggests that ILs may exhibit a degree of selectivity, demonstrating cytotoxic effects on cancer cells while sparing healthy cells at this concentration.

Moreover, the IC_50_ values of ILs for MCF-7 F cells are lower than those obtained for the standard chemotherapeutic agent Doxorubicin, which has an IC_50_ value of 7.20 µg/mL (13.21 µM). This underscores the potent inhibitory effect of ILs on cancer cell proliferation, potentially offering a promising alternative or adjunct to conventional therapies.

Overall, these findings emphasize the potential of ILs as selective anticancer agents, capable of effectively targeting cancer cells while sparing healthy tissues to some extent.

**Figure 12:**
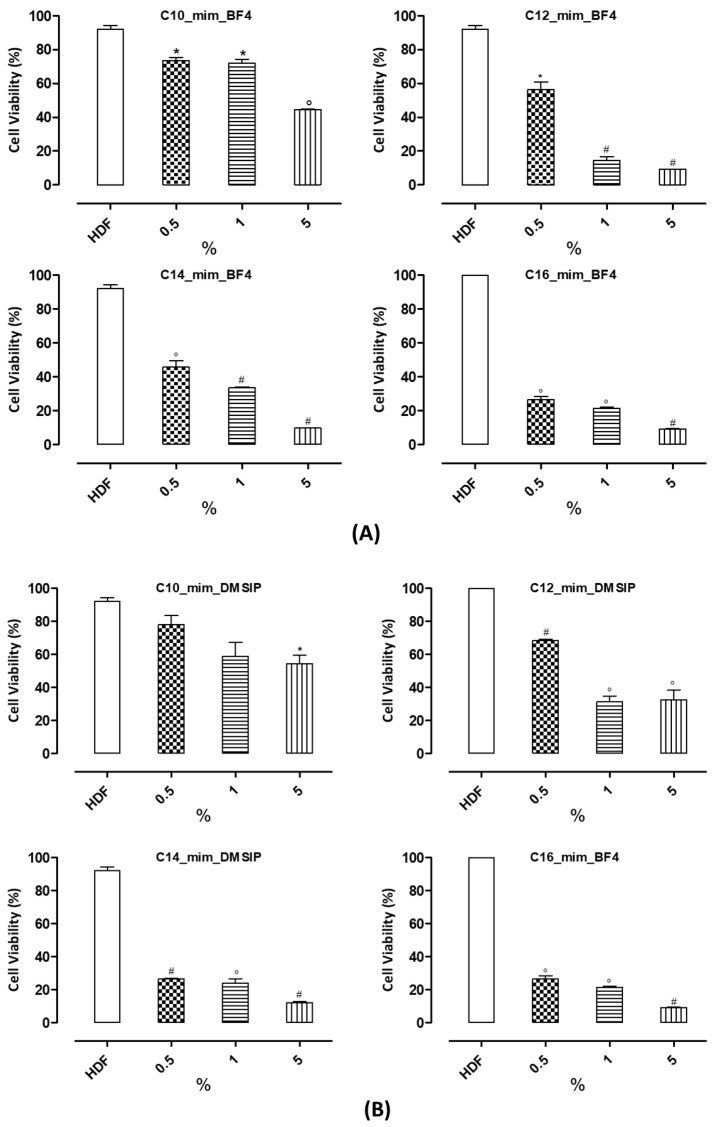
Indirect cytotoxic test. Alamar blue assay to evaluate the cell viability of (**A**) C*n*mimBF_4_-loaded PVC matrix (at different weight percentage, wt%) and (**B**) C*n*mimDMSIP-loaded PVC matrix (at different weight percentage, wt%) after 24 h of elution time and 24 h of in vitro cell culture * *p* < 0.01; ° *p* < 0.001; # *p* < 0.001 vs. HDF.

To assess the possibility of developing substrates for use in various biomedical applications, from drug delivery to tissue engineering or as an antimicrobial coating, the biocompatibility of ionic-liquid-loaded polymeric substrates (ILs) was also evaluated using indirect and direct cytotoxicity assays. This evaluation examined the effects of released chemicals and surface properties on cell viability. The results revealed that with C*n*mimBF_4_ (*n* = 10–16) loaded into the PVC matrix at various concentrations (0.5, 1, 5 wt%), cytotoxicity increased with higher quantity and alkyl chain lengths (Figure 12A). Notably, good cell survival occurred at 0.5 and 1 wt% concentrations of C10mimBF_4_. These findings suggest that ILs were released more rapidly into the cell culture medium, adversely affecting HDF cell viability. Similarly, the group loaded with C*n*mimDMSIP (*n* = 10–16) in the PVC matrix displayed a similar trend (Figure 12B).

**Figure 13:**
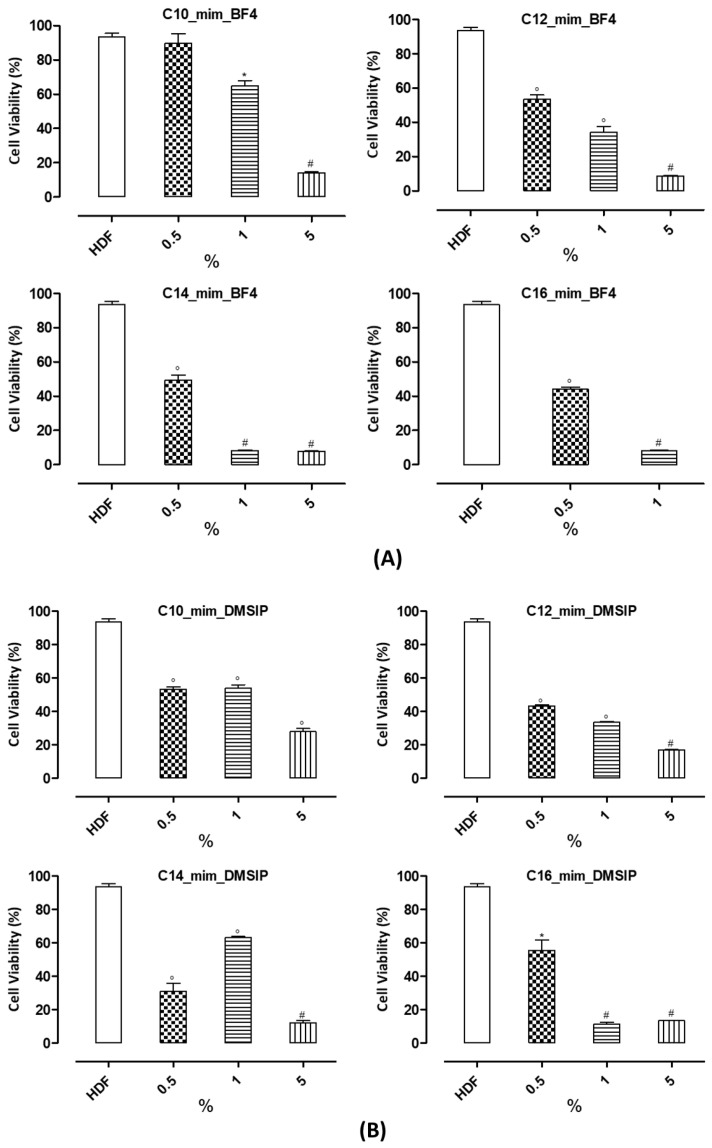
Direct cytotoxic test. Alamar blue assay to evaluate the cell viability of (**A**) C*n*mimBF_4_-loaded PVC matrix (at different weight percentage, wt%) and (**B**) C*n*mimDMSIP-loaded PVC substrates at direct contact with HDF cells (at different weight percentage, wt%). * *p* < 0.01; ° *p* < 0.001; # *p* < 0.001 vs. HDF.

**Figure 14:**
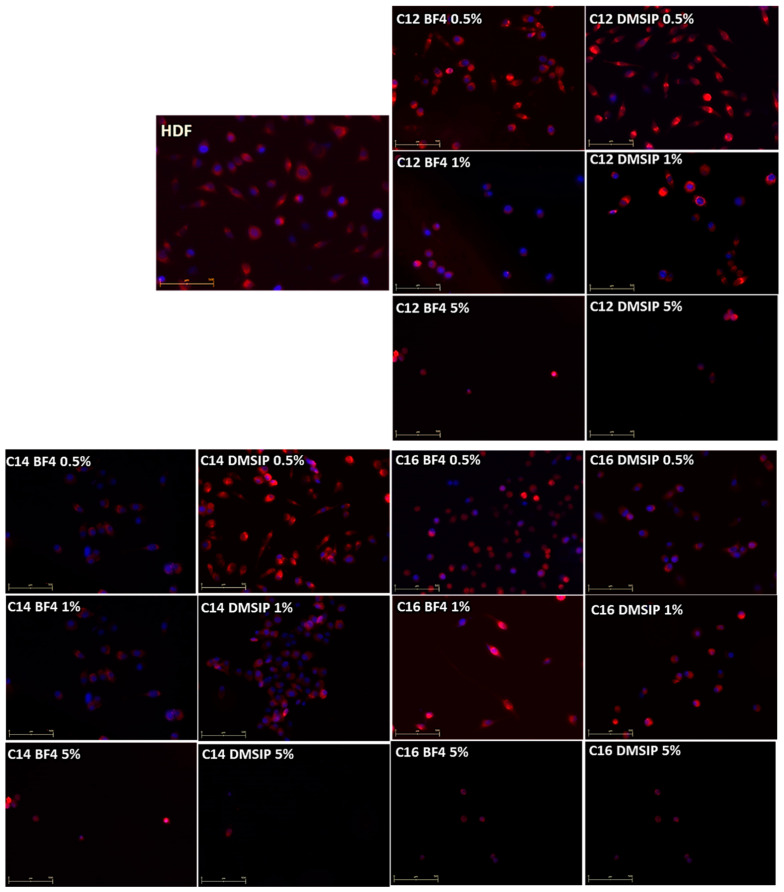
In vitro cell adhesion. Qualitative analyses of HDF cells stained with CellTracker™ Red CMTPX on C*n*mimBF_4_/C*n*mimDMSIP-loaded PVC substrates after 24 h of cell culture. Scale bar = 500 µm.

Furthermore, the IL-loaded polymer substrate underwent a direct cytotoxic assessment, with cell viability evaluated after 24 h of cell culture. The data shown in Figure 13 align consistently with those in Figure 14. Notably, increasing IL concentrations (wt%) decreased cell viability, with the observed impact potentially associated with the carbon chain lengths of the investigated ILs.

The quantitative findings align with the immunofluorescence assay conducted to assess the influence of material surfaces on cell activity in the initial hours of culture. The qualitative outcomes, depicted in Figure 14, demonstrate that material groups with reduced IL quantities and shorter alkyl chains display significantly enhanced cell viability. The biocompatibility assessment of IL-loaded PVC substrates provided valuable insights into their potential for biomedical applications. Both indirect and direct cytotoxicity assays were performed to evaluate the impact of released chemicals and surface properties on cell viability. Our results demonstrated that material groups with lower IL amounts and shorter alkyl chains exhibited notably high cell viability. This suggests that PVC composites with shorter alkyl chain ILs could be more suitable for biomedical applications where cell compatibility is crucial.

## 4. Conclusions

In this study, two series of methylimidazolium ionic liquids featuring long alkyl chains in their cations and tetrafluoroborate (BF_4_) and 1,3-dimethyl-5-sulfoisophthalate (DMSIP) as counterions were synthesized and characterised, and some of them were incorporated into the PVC matrix.

The thermal characterisation of neat ILs evidenced increasing thermal stability of both series with the elongation of the cation alkyl chains. Furthermore, better stability of the series containing the BF_4_ anion was found, besides indicating the role of the anion type in the ILs’ thermal behaviour. Preliminary studies on PVC/IL blends demonstrated the feasibility of producing antimicrobial films through an easy and cost-effective process, such as solvent casting. The PVC blends from both series of ILs showed a lowering of T_onset_ and T_d1_, which was not observed for the second step of degradation temperature (T_d2_). This decrease, likely due to ILs’ participation in the autocatalytic degradation of PVC, depends on both IL nature and concentration. Similarly, an IL dose-dependent reduction in the PVC hydrophobicity was determined, with a more substantial hydrophilic effect of the BF_4_ anion than that of DMSIP. Findings on IL release from PVC blends evidenced a higher and faster release of the ILs containing the BF_4_ anion. Moreover, the results demonstrated the potential to modulate the physicochemical properties of polymer matrices by varying IL concentrations, the alkyl chain length, and the type of anion.

On the biological front, the antimicrobial and antitumor activities of the pure ILs were primarily dependent on their structure. These activities were more pronounced in the series containing the BF_4_ anion and increased with the lengthening of the alkyl chain of the methylimidazolium cation. However, extending the alkyl chain beyond C16 decreased antimicrobial activity, indicating a cut-off effect.

The data indicating lower IC_50_ values for MCF-7 cells than healthy cells underscore the promising prospect of ILs as potential active agents in cancer therapy. Their ability to exhibit cytotoxic effects specifically on cancer cells while sparing healthy cells holds significant implications for targeted cancer treatment strategies. This selectivity not only minimizes the risk of adverse effects on healthy tissues but also enhances the therapeutic efficacy of ILs in combating cancer progression.

These findings pave the way for the further exploration of ILs as potential candidates for biomedical applications, particularly in the realm of cancer therapy. Harnessing the selective cytotoxicity of ILs against cancer cells could lead to the development of innovative and more efficacious anticancer treatments with reduced systemic toxicity. Future research endeavours aimed at elucidating the underlying mechanisms of this selectivity and optimising the therapeutic potential of ILs hold immense promise for advancing precision medicine approaches in cancer treatment.

Additionally, initial biological studies on PVC/IL blend films indicated the ability to modulate cellular response by adjusting the concentration and release of ILs from the polymeric blend. The produced PVC/IL films hold significant promise for various biomedical applications. Their versatility and compatibility make them suitable for various medical devices, including surgical implants and prosthetic devices. Furthermore, their antimicrobial properties make them ideal candidates for coating medical equipment, such as catheters and surgical instruments, to prevent infections. Overall, the potential applications of these PVC/IL films in the biomedical field are extensive and represent an exciting avenue for future research and development. However, their clinical use necessitates rigorous assessments in terms of biocompatibility, regulatory approval, and compatibility with drug formulations to ensure patient safety.

## Figures and Tables

**Table 1:** List of the alkyl substituents of the cation and the two counteranions of the ILs synthetised.

Cations	Anions
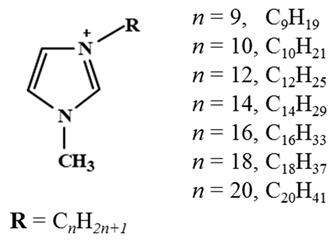	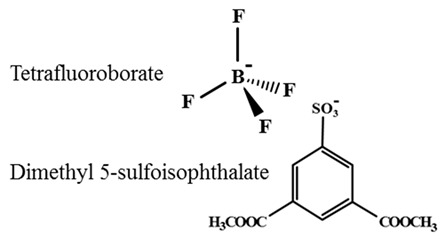

**Table 2:** Thermal degradation of neat PVC, PVC/C*n*mimBF_4_ and PVC/C*n*mimDMSIP film blends (*n* = 12, 14, 16).

Samples	T_Δm=5%_ (°C) ^a^	T_d1_ (°C) ^b^	T_d2_ (°C) ^c^	% R ^d^
Neat PVC	265	313	461	9
PVC/0.5 wt% C12mimBF_4_	231	241	463	7
PVC/1 wt% C12mimBF_4_	228	242	458	4
PVC/5 wt% C12mimBF_4_	216	227	459	5
PVC/0.5 wt% C14mimBF_4_	239	253	458	6
PVC/1 wt% C14mimBF_4_	238	252	463	6
PVC/5 wt% C14mimBF_4_	221	234	463	5
PVC/0.5 wt% C16mimBF_4_	238	253	460	6
PVC/1 wt% C16mimBF_4_	235	250	463	6
PVC/5 wt% C16mimBF_4_	225	238	466	6
PVC/0.5 wt% C12mimDMSIP	237	282	458	5
PVC/1 wt % C12mimDMSIP	235	255	460	9
PVC/5 wt % C12mimDMSIP	221	236	458	9
PVC/0.5 wt% C14mimDMSIP	242	291	459	5
PVC/1 wt% C14mimDMSIP	237	263	456	5
PVC/5 wt% C14mimDMSIP	226	244	459	8
PVC/0.5 wt% C16mimDMSIP	245	285	467	9
PVC/1 wt% C16mimDMSIP	244	282	464	9
PVC/5 wt% C16mimDMSIP	233	251	465	11

^a^ Temperature of 5% weight loss (onset temperature). ^b^ Decomposition maximum temperature (first step). ^c^ Decomposition maximum temperature (second step). ^d^ Weight residue (%) at 600 °C.

**Table 3:** Minimum inhibitory concentration (MIC) and minimum bactericidal concentration (MBC) of the alkyl methylimidazolium IL series containing BF_4_ and DMSIP as counter ions. *n* = C9, C10, C12, C14, C16, C18, C20. NI = no inhibition at IL maximum concentration used (1000 µM).

Antibacterial Activity µg/mL (µM)						
ILs	*Listeria monocytogenes*		*Escherichia coli*		*Pseudomonas fluorescens*	
	MIC	MBC	MIC	MBC	MIC	MBC
C9mimBF_4_	148.08 (500)	148.08 (500)	296.16 (1000)	296.16 (1000)	NI	NI
C10mimBF_4_	77.54 (250)	155.09 (500)	77.54 (250)	155.09 (500)	38.77 (125)	155.09 (500)
C12mimBF_4_	8.46 (25)	16.91(50)	16.91 (50)	84.56 (250)	16.91 (50)	42.28 (125)
C14mimBF_4_	4.58 (12.5)	4.58(12.5)	4.58 (12.5)	9.16 (25)	9.16 (25)	45.79 (125)
C16mimBF_4_	19.72 (50)	98.58 (250)	9.86 (25)	49.29 (125)	49.29 (125)	98.58 (250)
C18mimBF_4_	211.2 (500)	422.4 (1000)	52.80 (125)	105.60 (250)	NI	NI
C20mimBF_4_	NI	NI	NI	NI	NI	NI
C9mimDMSIP	NI	NI	482.59 (1000)	NI	NI	NI
C10mimDMSIP	124.16 (250)	496.62 (1000)	124.16 (250)	248.31 (500)	248.31 (500)	496.62 (1000)
C12mimDMSIP	13.12 (25)	26.24 (50)	26.24 (50)	65.58 (125)	65.58 (125)	262.33 (500)
C14mimDMSIP	27.64 (50)	69.09 (125)	13.82 (25)	27.64 (50)	27.64 (50)	69.09 (125)
C16mimDMSIP	29.04 (50)	72.60 (125)	72.60 (125)	72.60 (125)	145.19 (250)	145.19 (250)
C18mimDMSIP	304.4 (500)	304.4(500)	608.83 (1000)	608.83 (1000)	NI	NI
C20mimDMSIP	NI	NI	NI	NI	NI	NI

**Table 4:** Inhibition zones (mm, average of three replicates) determined by PVC loaded with increasing concentrations (0.5, 1, 5 wt%) of C*n*mimBF_4_ and C*n*mimDMSIP and neat PVC, for comparison, on NA plates spread with *L. monocytogenes*, *E. coli*, *P. fluorescens* at an initial concentration of 10^6^ CFU/mL and incubated for 24 h. NI—no inhibition.

Samples	*Listeria monocytogenes* Mean (mm) ± SD			*Escherichia coli* Mean (mm) ± SD			*Pseudomonas fluorescens* Mean (mm) ± SD		
	0.5%	1%	5%	0.5%	1%	5%	0.5%	1%	5%
PVC/C12mimBF_4_	NI	NI	NI	NI	NI	3.7 ± 1.2	NI	NI	NI
PVC/C14mimBF_4_	1.3 ± 0.3	2.7 ± 0.4	6.0 ± 1.0	NI	NI	2.7 ± 0.7	NI	1.7 ± 0.7	4.3 ± 1.2
PVC/C16mimBF_4_	NI	1.0 ± 0.3	2.0 ± 0.5	NI	NI	NI	NI	NI	NI
PVC/C12mimDMSIP	NI	NI	NI	NI	NI	3.3 ± 0.6	NI	NI	2.0 ± 1.0
PVC/C14mimDMSIP	NI	1.3 ± 0.3	2.5 ± 0.5	NI	NI	2.7 ± 1.0	NI	NI	2.5 ± 0.5
PVC/C16mimDMSIP	NI	NI	2.0 ± 0.5	NI	NI	NI	NI	NI	1.0 ± 0.3

**Table 5:** IC_50_ values of ILs for MCF7 cancer cells and HDF cells after 24 h of incubation. IC_50_ values were calculated using Grapad Prism 5 software, representing the mean of at least 3 independent experiments (*n* = 3, mean ± SD).

Samples	MCF7 µg/mL (µM)	HDF µg/mL (µM)
C9mimBF_4_	>14.81 (>50)	5.14 ± 0.10 (17.355)
C9mimDMSIP	>24.13 (>50)	4.96 ± 0.05 (10.28)
C10mimBF_4_	7.39 (23.82 ± 1.52)	4.18 ± 0.03 (13.48)
C10mimDMSIP	5.42 (10.91 ± 1.18)	5.56 ± 0.04 (11.196)
C12mimBF_4_	2.05 (6.062 ± 1.15)	4.34 ± 0.01 (12.83)
C12mimDMSIP	4.95 (9.44 ± 1.14)	5.56 ± 0.04 (10.597)
C14mimBF_4_	4.41 (12.05 ± 1.42)	3.78 ± 0.17 (10.32)
C14mimDMSIP	2.91 (5.22 ± 1.43)	5.56 ± 0.04 (10.059)
C16mimBF_4_	1.64 (4.16 ± 1.07)	8.26 ± 0.03 (20.95)
C16mimDMSIP	3.84 (6.61 ± 1.41)	5.56 ± 0.04 (9.57)
C18mimBF_4_	1.95 (4.61 ± 1.06)	4.57 ± 0.06 (10.82)
C18mimDMSIP	1.96 (3.22 ± 1.07)	4.01 ± 0.14 (6.59)
C20mimBF_4_	3.47 (7.71 ± 1.27)	2.70 ± 0.19 (5.99)
C20mimDMSIP	4.71 (7.39 ± 1.41)	5.10 ± 0.02 (8.008)

## Data Availability

The data presented in this study are available on request from the corresponding author.

## Supplementary Materials

The following supporting information can be downloaded at https://www.mdpi.com/article/10.3390/pharmaceutics16050642/s1, Table S1: molecular weight, color, physical state, T_m_ and ^1^H-NMR spectral data of the ILs synthesized; Table S2: formulas, calculated and measured *m*/*z* values of the peaks assigned to cations and adducts of ILs by MALDI TOF analysis; Table S3: ILs release (%) from the PVC blends loaded with the PVC/C*n*mimBF_4_ and PVC/C*n*mimDMSIP blends, at different concentrations (0.5, 1, 5%); Figure S1: ^1^H-NMR spectrum (400 MHz, DMSO-*d*_6_, δ ppm) of the IL 1-dodecyl-3-methylimidazolium tetrafluoroborate (C12mimBF_4_); Figure S2: ^1^H-NMR spectrum (400 MHz, DMSO-*d*_6_, δ ppm) of the IL 1-dodecyl-3-methylimidazolium 1,3-dimethyl-5-sulfoisophthalate (C12mimDMSIP); Figure S3: Images of neat PVC and PVC blend films loaded with 5 wt% concentration of C12mimDMSIP (A); C14mimDMSIP (B), C16mimDMSIP (C) and C12mimBF_4_ (D), C14mimBF_4_ (E), C16mimBF_4_ (F); Figure S4: DSC curves of neat PVC TOTM; Figure S5: DSC curves of neat PVC TOTM and PVC/C*n*mimBF_4_ blend films (*n* = 12, 14, 16); Figure S6: DSC curves of neat PVC and PVC/C*n*mimDMSIP blend films (*n* = 12, 14, 16); Figure S7: SEM picture (magnification 2000×) of neat PVC; Figure S8: SEM pictures (magnification 1000×) of PVC blend films containing the 0.5 wt% of (A) C12mimBF_4_, (B) C14mimBF_4_, (C) C16mimBF_4_, (D) C12mimDMSIP, (E) C14mimDMSIP, (F) C16mimDMSIP.

## Abbreviations

**Ionic Liquids**	
Cations	
C9mim	1-methyl-3-nonylimidazolium
C10mim	1-decyl-3-methylimidazolium
C12mim	1-dodecyl-3-methylimidazolium
C14mim	1-methyl-3-tetradecylimidazolium
C16mim	1-hexyl-3-methylimidazolium
C18mim	1-methyl-3-octadecylimidazolium
C20mim	1-eicosyl-3-methylimidazolium
Anions	
BF_4_	tetrafluoroborate
DMSIP	1,3-dimethyl 5-sulfoisophthalate
**Bacteria**	
*L. monocytogenes*	*Listeria monocytogenes*
*E. coli*	*Escherichia coli*
*P. fluorescens*	*Pseudomonas fluorescens*
**Human cell lines**	
MCF7	human breast cancer cells
HDF	human dermal fibroblast cells
