# Supplementary material for: Synthesis, Characterisation, and In Vitro Evaluation of Biocompatibility, Antibacterial and Antitumor Activity of Imidazolium Ionic Liquids
Source: Pharmaceutics. 2024 May 10;16(5):642. doi: 10.3390/pharmaceutics16050642 (PMC11125126; doi:10.3390/pharmaceutics16050642)
Supplement: Supplementary file 1 [file pharmaceutics-16-00642-s001.zip › pharmaceutics-2965208-supplementary.pdf]

Synthesis, Characterisation, and In Vitro Evaluation of Biocompatibility, Antibacterial and Antitumor Activity of Imidazolium Ionic Liquids

Elisabetta Novello [1,†], Giuseppina Scalzo [1,†], Giovanni D'Agata [1], Maria G. Raucci [2,*], Luigi Ambrosio [2], Alessandra Soriente [2], Barbara Tomasello [3], Cristina Restuccia [4], Lucia Parafati [4], Grazia M. L. Consoli [5], Loredana Ferreri [5], Antonio Rescifina [3], Chiara Zagni [3,*] and Daniela C. Zampino [1]

[1] *Institute of Polymers, Composites and Biomaterials (IPCB)—CNR, Section of Catania, Via Paolo Gaifami, 18, 95126 Catania, Italy*

[2] *Institute of Polymers, Composites and Biomaterials (IPCB)—CNR, Section of Napoli, Viale J.F. Kennedy n.54, Pad.20, 80125 Napoli, Italy*

[3] *Department of Drug and Health Sciences, University of Catania, Viale A. Doria 6, 95125 Catania, Italy*

[4] *Department of Agriculture, Food and Environment, University of Catania, Via Santa Sofia 100, 95123 Catania, Italy*

[5] *Institute of Biomolecular Chemistry (ICB)-CNR, via Paolo Gaifami 18, 95126 Catania, Italy*

* Corresponding authors:
Maria G. Raucci, E-mail Address: mariagrazia.raucci@cnr.it
Chiara Zagni, E-mail Address: chiara.zagni@unict.it

† These authors have contributed equally to the research

IL synthesis

The ILs were synthesized using a two-step method involving the alkylation of the imidazolium ring and metathesis reactions, slightly modifying previously reported synthetic procedures [1-3].

The IL series containing the Br anion (C9mimBr, C10mimBr, C12mimBr, C14mimBr, C16mimBr, C18mimBr, C20mImBr) was synthesized by reacting equimolar amounts of 1-methylimidazole with alkyl bromides containing C9, C10, C12, C14, C16, C18 and C20 hydrocarbon chains, respectively. The reactions were performed at 60 °C for 24 h under a nitrogen atmosphere and vigorous stirring. The obtained products were washed with ethyl acetate and dried in a vacuum oven for 24–48 h.

The ionic exchange between the synthesized 1-alkyl-3-methylimidazolium bromides and the sodium salts of tetrafluoroborate (BF_4) and 1,3-dimethyl-5-sulfoisophthalate (DMSIP) was made by stirring the solutions containing the alkyl-methylimidazolium bromides (0.020 mol) in DCM and the chosen salts (0.021 mol) in water at room temperature (RT) for 1 h. Then, the obtained solutions were transferred together into a separating funnel and stirred vigorously. When both phases became clear, well separated and without precipitates, the organic layer was taken, dried over anhydrous sodium sulphate and filtrated. The resulting viscous-liquid ILs (C9mimBF4, C9mimDMSIP, C10mimBF_4, C10mimDMSIP) were dried at 25–30 °C for 48 h, whereas the powders of the other ILs were dried in a vacuum drying oven for 48 h at 40–45 ºC. The complete exchange of the bromide counter-ion was verified by the silver nitrate test. If the exchange was not completed, a new water solution containing the same sodium salt was added to the organic phase to obtain a complete exchange.

Synthesis of the C*n*mimBF_4 series

1-nonyl-3-methylimidazolium tetrafluoroborate (C9mimBF_4)

1-nonyl-3-methylimidazolium bromide (0.020 mol, 5.78 g) was dissolved in 50 mL of DCM and added to 50 mL water solution of sodium tetrafluoroborate (0.022 mol, 2.41 g) in a separating funnel. The reaction proceeds according to the metathesis procedure. A yellow viscous oil was obtained. Yield 70%.

1-decyl-3-methylimidazolium tetrafluoroborate (C10mimBF_4)

1-decyl-3-methylimidazolium bromide (20 mmol, 6.04 g) was dissolved in 50 mL of DCM and added to 50 mL water solution of sodium tetrafluoroborate (22 mmol, 2.41 g) in a separating funnel. The reaction proceeds according to the metathesis procedure. A yellow viscous oil was obtained. Yield 72%.

1-dodecyl-3-methylimidazolium tetrafluoroborate ($C12mimBF_4$)

1-dodecyl-3-methylimidazolium bromide (20 mmol, 6.60 g) was dissolved in 50 mL of DCM and added to 50 mL water solution of sodium tetrafluoroborate (22 mmol, 2.41 g) in a separating funnel. The reaction proceeds according to the metathesis procedure. A white solid was obtained. Yield 82%.

1-tetradecyl-3-methylimidazolium tetrafluoroborate ($C14mimBF_4$)

1-tetradecyl-3-methylimidazolium bromide (20 mmol, 7.16 g) was dissolved in 50 mL of dichloromethane (DCM) and added to a solution of sodium tetrafluoroborate salt (22 mmol, 2.41 g) dissolved in 50 mL of water in a separating funnel. The reaction proceeds according to the procedure used for metathesis 2. A white solid was obtained. Yield 88%.

1-hexadecyl-3-methylimidazolium tetrafluoroborate ($C16mimBF_4$)

1-hexadecyl-3-methyl-imidazolium bromide (20 mmol, 7.74 g) was dissolved in 50 ml of DCM and added to a solution of sodium tetrafluoroborate (22 mmol, 2.41 g) dissolved in 50 mL of water in a separating funnel. The reaction proceeds according to the procedure used for metathesis 2. A white solid was obtained. Yield 80%.

1-octadecyl-3-methylimidazolium tetrafluoroborate ($C18mimBF_4$)

1-octadecyl-3-methylimidazolium bromide (20 mmol, 8.30 g) was dissolved in 50 mL of DCM and added to a solution of sodium tetrafluoroborate (22 mmol, 2.41 g) dissolved in 50 mL of water in a separating funnel. The reaction proceeds according to the procedure used for metathesis 2. A light-yellow solid was obtained. Yield 85%.

1-eicosyl-3-methylimidazolium tetrafluoroborate ($C20mimBF_4$)

1-eicosyl-3-methylimidazolium bromide (20 mmol, 8.87 g) was dissolved in 50 ml of DCM and added to a solution of sodium tetrafluoroborate (22 mmol, 2.41 g) dissolved in 50 mL of water in a separating funnel. The reaction proceeds according to the procedure used for metathesis 2. A pale-yellow solid was obtained. Yield 84%.

Synthesis of the C*n*mimDMSIP series

1-nonyl-3-methylimidazolium 1,3-dimethyl-5-sulfoisophthalate (C9mimDMSIP)

1-nonyl-3-methylimidazolium bromide (0.020 mol, 5.78 g) was dissolved in 50 mL of DCM and added to 50 mL water solution of sodium tetrafluoroborate (0.022 mol, 6.51 g) in a separating funnel. The reaction proceeds according to the metathesis procedure. A yellow viscous oil was obtained. Yield 73%.

1-decyl-3-methylimidazolium 1,3-dimethyl-5-sulfoisophthalate (C10mimDMSIP)

1-decyl-3-methylimidazolium bromide (20 mmol, 6.04 g) was dissolved in 50 mL of DCM and added to 50 mL water solution of sodium tetrafluoroborate (22 mmol, 6.51 g) in a separating funnel. The reaction proceeds according to the metathesis procedure. A yellow viscous oil was obtained. Yield 75%.

1-dodecyl-3-methylimidazolium 1,3-dimethyl-5-sulfoisophthalate (C12mimDMSIP)

1-dodecyl-3-methylimidazolium bromide (20 mmol, 6.60 g) was dissolved in 50 mL of DCM and added to 50 mL water solution of sodium tetrafluoroborate (22 mmol, 6.51 g) in a separating funnel. The reaction proceeds according to the metathesis procedure. A white solid was obtained. Yield 80%.

1-tetradecyl-3-methylimidazolium 1,3-dimethyl-5-sulfoisophthalate (C14mimDMSIP)

1-tetradecyl-3-methylimidazolium bromide (20 mmol, 7.16 g) was dissolved in 50 mL of DCM and added to a solution of sodium dimethyl-5-sulfoisophthalate (22 mmol, 6.51 g) dissolved in 50 mL of water in a separating funnel. The reaction proceeds according to the procedure used for metathesis 2. A white solid was obtained. Yield 85%.

1-hexadecyl-3-methylimidazolium 1,3-dimethyl-5-sulfoisophthalate (C16mimDMSIP)

1-hexadecyl-3-methylimidazolium bromide (20 mmol, 7.74 g) was dissolved in 50 mL of DCM and added to a solution of sodium dimethyl-5-sulfoisophthalate (22 mmol, 6.51 g) dissolved in 50 mL of water in a separating funnel. The reaction proceeds according to the procedure used for metathesis 2. A white solid was obtained. Yield 93%.

1-octadecyl-3-methylimidazolium 1,3-dimethyl-5-sulfoisophthalate (C18mimDMSIP)

1-octadecyl-3-methylimidazolium bromide (20 mmol, 8.30 g) was dissolved in 50 mL of DCM and added to a solution of sodium dimethyl-5-sulfoisophthalate (22 mmol, 6.51 g) dissolved in 50 mL of

water in a separating funnel. The reaction proceeds according to the procedure used for metathesis 2. A light-yellow solid was obtained. Yield 82%.

1-eicosyl-3-methylimidazolium 1,3-dimethyl-5-sulfoisophthalate (C20mimDMSIP)

1-eicosyl-3-methylimidazolium bromide (20 mmol, 8.87 g) was dissolved in 50 mL of DCM and added to a solution of sodium dimethyl-5-sulfoisophthalate (22 mmol, 6.51 g) dissolved in 50 mL of water in a separating funnel. The reaction proceeds according to the procedure used for metathesis 2. A pale-yellow solid was obtained. Yield 82%.

Characterisation of pure ILs, neat PVC and PVC/IL films

Table S1. Molecular weight, color, physical state, T_m and ^{1}H-NMR spectral data of the ILs synthesized.

Table S2. Formulas, calculated and measured m/z values of the peaks assigned to cations and adducts of ILs by MALDI TOF analysis.

Table S3. ILs release (%) from the PVC blends loaded with the PVC/C*n*mimBF$_4$ and PVC/C*n*mimDMSIP blends, at different concentrations (0.5, 1, 5%).

Figure S1. ^{1}H-NMR spectrum (400 MHz, DMSO-d$_6$, δ ppm) of the IL 1-dodecyl-3-methylimidazolium tetrafluoroborate (C12mimBF$_4$).

Figure S2. ^{1}H-NMR spectrum (400 MHz, DMSO-d$_6$, δ ppm) of the IL 1-dodecyl-3-methylimidazolium 1,3-dimethyl-5-sulfoisophthalate (C12mimDMSIP).

Figure S3. Images of neat PVC and PVC blend films loaded with 5 wt% concentration of C12mimDMSIP (A); C14mimDMSIP (B), C16mimDMSIP (C) and C12mimBF$_4$ (D), C14mimBF$_4$ (E), C16mimBF$_4$ (F).

Figure S4. DSC curves of neat PVC TOTM.

Figure S5. DSC curves of neat PVC TOTM and PVC/C*n*mimBF$_4$ blend films (n=12, 14, 16).

Figure S6. DSC curves of neat PVC and PVC/C*n*mimDMSIP blend films (n=12, 14, 16).

Figure S7. SEM picture (magnification 2000x) of neat PVC

Figure S8. SEM pictures (magnification 1000x) of PVC blend films containing the 0.5 wt% of (A) C12mimBF$_4$, (B) C14mimBF$_4$, (C) C16mimBF$_4$, (D) C12mimDMSIP, (E) C14mimDMSIP, (F) C16mimDMSIP.

Table S1. Molecular weight, colour and physical state and T_m of the ILs synthesized, together with their ^{1}H-NMR spectral data. The chemical shift of the C*n*mimBF$_4$ series and the IL C16mimDMSIP, obtained by using deuterated acetone, CDCl$_3$/TFA and CDCl$_3$, were previously reported in the literature [1-3].

**ILs**	**Mw** g/mol	**Colour/State Yield**	**T_m (°C)**	**^{1}H-NMR Chemical Shift (400 MHz, DMSO-d$_6$, δ ppm)**
C_9mimBF$_4$	296.16	Yellow/viscous oil 70%	-	0.86 (t, 3H, CH_3-C_8), 1.25 (m, 12 H, CH_2), 1.77 (m, 2H, CH_2-CH_2-N) 3.84 (s, 3H, CH_3-N) 4.15 (t, 2H, CH_2-N), 7.70 (s, 1H, CH in imidazolium ring), 7.77 (s, 1H, CH in imidazolium ring), 9.09 (s, 1H, CH in imidazolium ring).
C_9mimDMSIP	482.59	Yellow/viscous oil 73%	-	0.85 (t, 3H, CH3-C8), 1.23 (m, 12 H, CH2), 1.76 (m, 2H, CH2-CH2-N) 3.84 (s, 3H, CH3-N) 4.14 (t, 2H, CH2-N), 7.69 (s, 1H, CH in imidazolium ring), 7.76 (s, 1H, CH in imidazolium ring), 9.09 (s, 1H, CH in imidazolium ring).<br>Signals of the benzene ring in 1,3-dimethyl -5-sulfoisophthalate anion: 3.90 (s, 6H, CH_3-O), 8.39 (d, 2H, CH in ortho position with respect to SO_3^- substituents), 8.42 (d, 1H, CH in para position with respect to SO_3^- substituents).
C_{10}mimBF$_4$	310.18	Yellow/viscous oil 72%	--	0.85 (t, 3H, CH_3-C_9), 1.24 (m, 14 H, CH_2), 1.77 (m, 2H, CH_2-CH_2-N) 3.84 (s, 3H, CH_3-N) 4.14 (t, 2H, CH_2-N), 7.69 (s, 1H, CH in imidazolium ring), 7.76 (s, 1H, CH in imidazolium ring), 9.09 (s, 1H).
C_{10}mimDMSIP	496.62	Yellow/viscous oil 75%	-	Signals of the benzene ring in dimethyl-5-sulfoisophthalate anion: 3.91 (s, 6H, CH_3-O), 8.39 (d, 2H, CH in ortho position with respect

				to SO_3^- substituents), 8.42 (d, 1H, CH in para position with respect to SO_3^- substituents). Signals of the benzene ring in 1,3-dimethyl-5-sulfoisophthalate anion: 3.91 (s, 6H, CH_3-O), 8.39 (d, 2H, CH in ortho position with respect to SO_3^- substituents), 8.42 (d, 1H, CH in para position with respect to SO_3^- substituents).
C_{12}mimBF$_4$	338.24	White solid 82%	31	0.86 (t, 3H, CH_3-C_{11}), 1.24 (m, 18 H, CH_2), 1.77 (m, 2H, CH_2-CH_2-N) 3.84 (s, 3H, CH_3-N) 4.14 (t, 2H, CH_2-N), 7.70 (s, 1H, CH in imidazolium ring), 7.77 (s, 1H, CH in imidazolium ring), 9.10 (s, 1H, CH in imidazolium ring).
C_{12}mimDMSIP	524.67	White solid 80%	-	0.84 (t, 3H, CH_3-C_{11}), 1.22 (m, 18 H, CH_2), 1.75 (m, 2H, CH_2-CH_2-N) 3.83 (s, 3H, CH_3-N) 4.13 (t, 2H, CH_2-N), 7.69 (s, 1H, CH in imidazolium ring), 7.76 (s, 1H, CH in imidazolium ring), 9.10 (s, 1H, CH in imidazolium ring). Signals of the benzene ring in 1,3-dimethyl-5-sulfoisophthalate anion: 3.90 (s, 6H, CH_3-O), 8.38 (d, 2H, CH in ortho position with respect to SO_3^- substituents), 8.42 (d, 1H, CH in para position with respect to SO_3^- substituents).
C_{14}mimBF$_4$	366.29	White solid 88%	39	0.85 (t, 3H), 1.23 (m, 22H), 1.76 (m, 2H), 3.84 (s, 3H), 4.14 (t, 2H), 7.69 (s, 1H), 7.76 (s, 1H), 9.09 (s, 1H).
C_{14}mimDMSIP	552.72	White solid 85%	-	0.85 (t, 3H), 1.23 (m, 22H), 1.76 (m, 2H), 3.84 (s, 3H), 4.14 (t, 2H), 7.70 (s, 1H), 7.76 (s, 1H), 9.10 (s, 1H).

				Signals of the benzene ring in 1,3-dimethyl-5-sulfoisophthalate anion: 3.91 (s, 6H, CH_3-O), 8.39 (d, 2H, CH in ortho position with respect to SO_3^- substituents), 8.42 (d, 1H, CH in para position with respect to SO_3^- substituents).
$C_{16}mimBF_4$	394.34	White solid<br>80%	50	0.85 (t, 3H), 1.23 (m, 26H), 1.76 (m, 2H), 3.84 (s, 3H), 4.14 (t, 2H), 7.69 (s, 1H), 7.76 (s, 1H), 9.08 (s, 1H).
C_{16}mimDMSIP	580.78	White solid<br>93%	49	0.85 (t, 3H), 1.23 (m, 26H), 1.76 (m, 2H), 3.84 (s, 3H), 4.14 (t, 2H), 7.70 (s, 1H), 7.77 (s,1H), 9.10 (s, 1H).<br>Signals of the benzene ring in 1,3-dimethyl-5-sulfoisophthalate anion: 3.91 (s, 6H, CH_3-O), 8.39 (d, 2H, CH in ortho position with respect to SO_3^- substituents), 8.42 (d, 1H, CH in para position with respect to SO_3^- substituents).
$C_{18}mimBF_4$	422.40	Light-yellow solid<br>85%	59	0.85 (t, 3H), 1.23 (m, 30H), 1.76 (m, 2H), 3.84 (s, 3H), 4.14 (t, 2H), 7.69 (s, 1H), 7.76 (s, 1H), 9.09 (s, 1H).
C_{18}mimDMSIP	608.83	Light-yellow solid<br>82%	62	0.84 (t, 3H), 1.22 (m, 30H), 1.75 (m, 2H), 3.84 (s, 3H), 4.13 (t, 2H), 7.70 (s, 1H), 7.76 (s, 1H), 9.11 (s, 1H).<br>Signals of the benzene ring in 1,3-dimethyl-5-sulfoisophthalate anion: 3.90 (s, 6H, CH_3-O), 8.39 (d, 2H, CH in ortho position with respect to SO_3^- substituents), 8.42 (d, 1H, CH in para position with respect to SO_3^- substituents).

$C_{20}mimBF_4$	450.45	Pale-yellow solid 84%	70	0.85 (t, 3H), 1.23 (m, 34H), 1.76 (m, 2H), 3.84 (s, 3H), 4.14 (t, 2H), 7.69 (s, 1H), 7.76 (s, 1H), 9.09 (s, 1H).
$C_{20}mimDMSIP$	636.88	Pale-yellow solid 82%	70	0.85 (t, 3H), 1.22 (m, 34H), 1.76 (m, 2H), 3.84 (s, 3H), 4.14 (t, 2H), 7.69 (s, 1H), 7.76 (s, 1H), 9.09 (s, 1H). Signals of the benzene ring in 1,3-dimethyl-5-sulfoisophthalate anion: 3.91 (s, 6H, CH_3-O), 8.39 (d, 2H, CH in ortho position with respect to SO_3^- substituents), 8.42 (d, 1H, CH in para position with respect to SO_3^- substituents).

Table S2. Formulas, calculated and measured *m/z* values of the peaks assigned to cations and adducts of ILs by MALDI TOF analysis. The adducts are constituted by two cations and the anion.

**Cations**			**IL Adducts (BF_4)**			**IL Adducts (DMSIP)**		
Formulas	***Measured m/z***	***Calculated m/z***	***Formulas***	***Measured m/z***	***Calculated m/z***	***Formulas***	***Measured m/z***	***Calculated m/z***
**1-methyl-3-nonylimidazole [C9mim]$^+$**			$[(C9mim)_2]^+ BF_4^-$			$[(C9mim)_2]^+$ DMSIP$^-$		
$C_{13}H_{25}N_2^+$	209.19	209.20	$C_{26}H_{50}N_4BF_4^+$	505.49	505.41	$C_{36}H_{59}N_4O_7S^+$	691.56	691.41
**1-decyl-3-methylimidazole [C10mim]$^+$**			$[(C10mim)_2]^+ BF_4^-$			$[(C10mim)_2]^+$ DMSIP$^-$		
$C_{14}H_{27}N_2^+$	223.20	223.22	$C_{28}H_{54}N_4BF_4^+$	533.53	533.44	$C_{38}H_{63}N_4O_7S^+$	719.59	719.44
**1-dodecyl-3-methylimidazole [C12mim]$^+$**			$[(C12mim)_2]^+ BF_4^-$			$[(C12mim)_2]^+$ DMSIP$^-$		
$C_{16}H_{31}N_2^+$	251.28	251.25	$C_{32}H_{62}N_4BF_4^+$	589.59	589.50	$C_{42}H_{71}N_4O_7S^+$	775.63	775.50
**1-methyl-3-tetradecylimidazolium [C14mim]$^+$**			$[(C14mim)_2]^+ BF_4^-$			$[(C14mim)_2]^+$ DMSIP$^-$		
$C_{18}H_{35}N_2^+$	279.25	279.28	$C_{36}H_{70}N_4BF_4^+$	645.55	645.56	$C_{46}H_{79}N_4O_7S^+$	832.56	832.57
**1-hexadecyl-3-methylimidazolium [C16mim]$^+$**			$[(C16mim)_2]^+ BF_4^-$			$[(C16mim)_2]^+$ DMSIP$^-$		
$C_{20}H_{39}N_2^+$	307.34	307.31	$C_{40}H_{78}N_4BF_4^+$	701.82	701.63	$C_{50}H_{87}N_4O_7S^+$	887.77	887.63
**1-methyl-3-octadecylimidazolium [C18mim]$^+$**			$[(C18mim)_2]^+ BF_4^-$			$[(C18mim)_2]^+$ DMSIP$^-$		
$C_{22}H_{43}N_2^+$	335.35	335.59	$C_{44}H_{86}N_4BF_4^+$	757.65	757.69	$C_{54}H_{95}N_4O_7S^+$	757.65	757.69
**1-eicosyl-3-methylimidazolium [C20mim]$^+$**			$[(C20mim)_2]^+ BF_4^-$			$[(C20mim)_2]^+$ DMSIP$^-$		
$C_{24}H_{47}N_2^+$	363.27	363.64	$C_{48}H_{94}N_4BF_4^+$	813.68	813.75	$C_{58}H_{103}N_4O_7S^+$	999.69	999.75

Table S3. IL release (%) from the PVC blends loaded with the PVC/C*n*mimBF_4 and PVC/C*n*mimDMSIP blends, at different concentrations (0.5, 1, 5%). The release (%) values are calculated from the amounts of ILs released after 24 h.

**Samples**	**IL content**		
	**0.5%**	**1%**	**5%**
PVC/C12mimBF_4	5.9	5.1	2.6
PVC/C14mimBF_4	23.8	24.2	30.4
PVC/C16mimBF_4	18.5	28.2	29.6
PVC/C12mimDMSIP	4.2	3.2	1.8
PVC/C14mimDMSIP	6.3	6.7	6.9
PVC/C16mimDMSIP	6.5	9.7	16.3

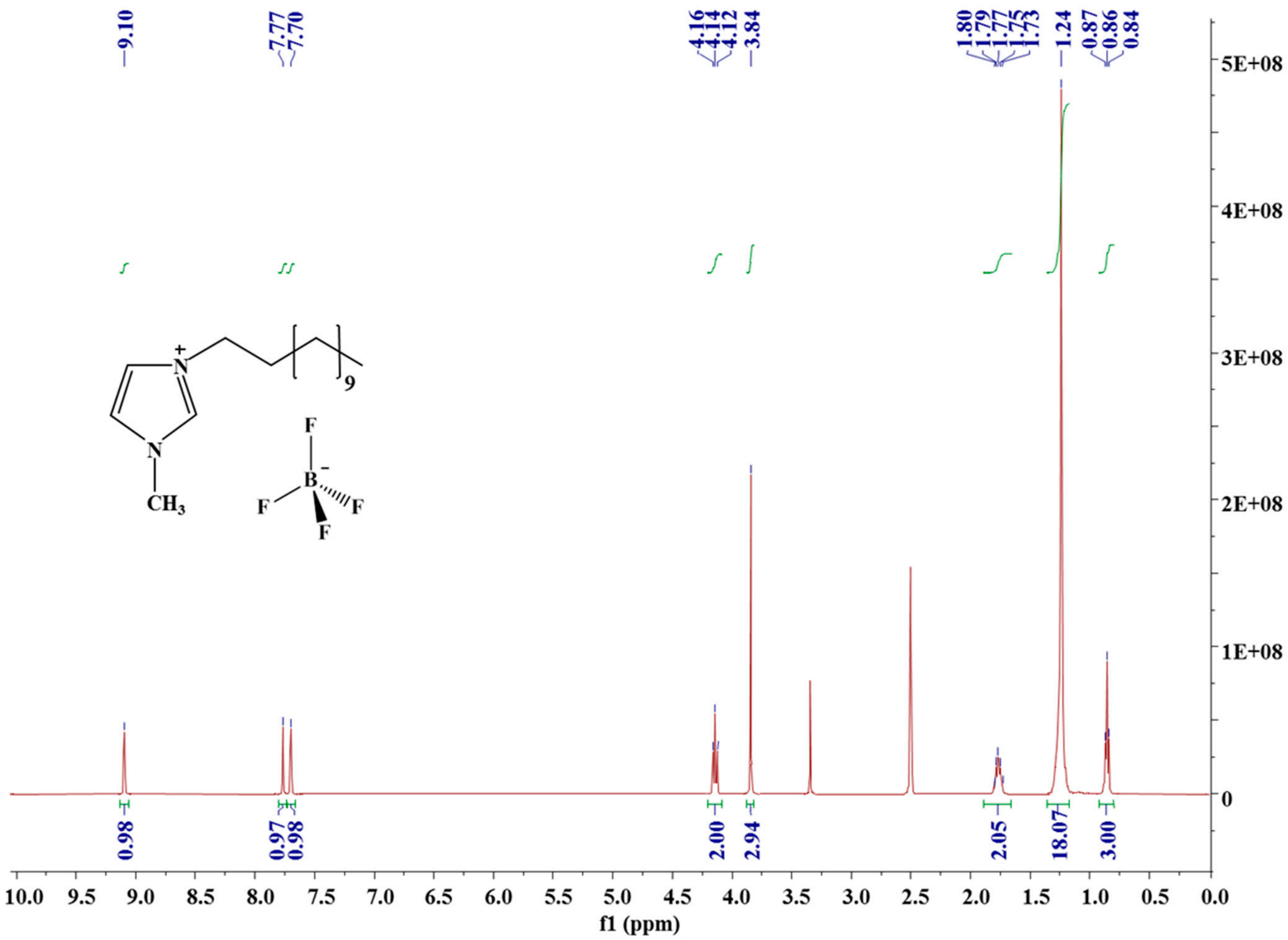

Figure S1. ^{1}H-NMR spectrum (400 MHz, DMSO-d_6, δ ppm) of the IL 1-dodecyl-3-methylimidazolium tetrafluoroborate ($C12mimBF_4$)

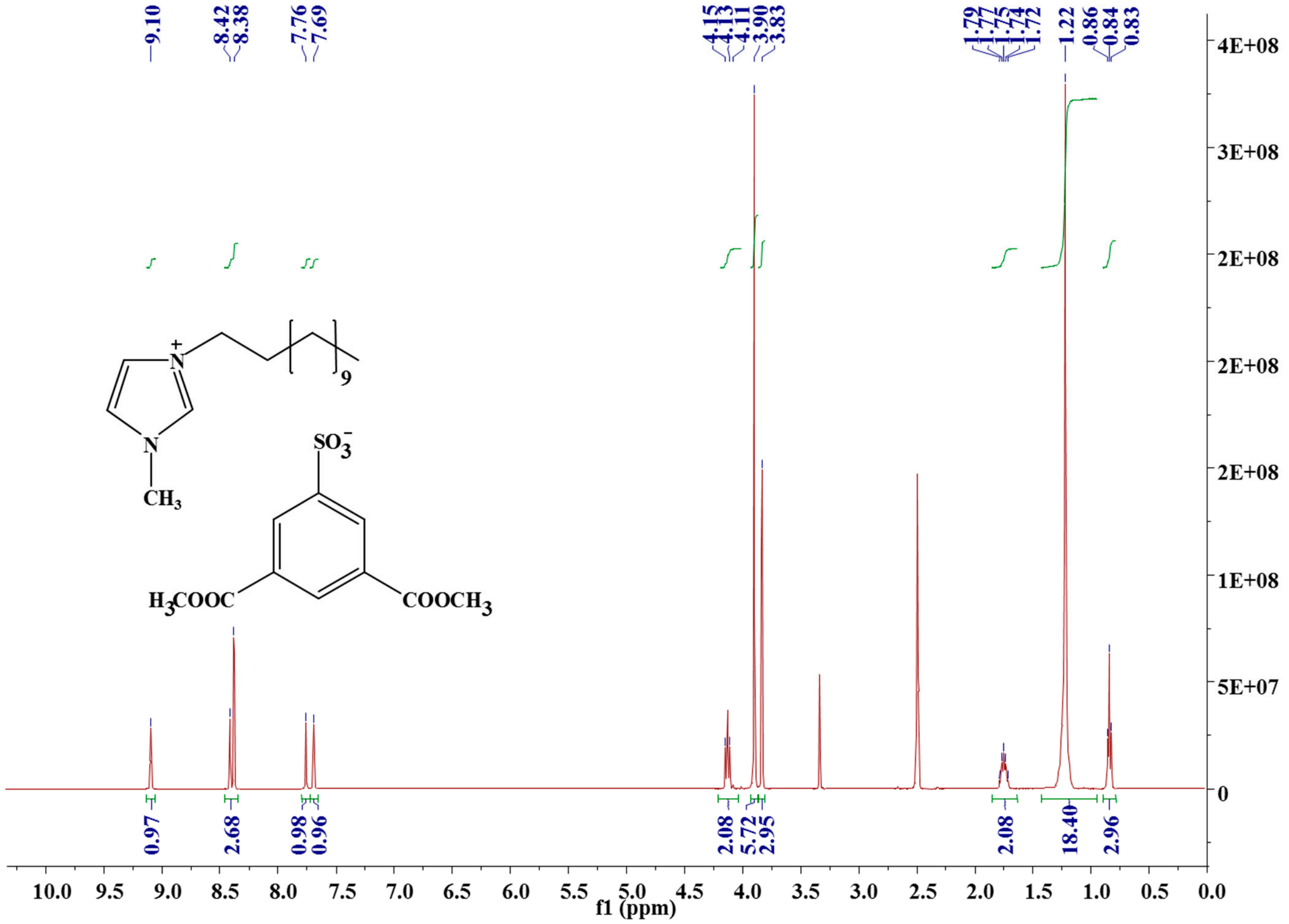

Figure S2. ^{1}H-NMR spectrum (400 MHz, DMSO-d_6, δ ppm) of the IL 1-dodecyl-3-methylimidazolium 1,3-dimethyl-5-sulfoisophthalate (C12mimDMSIP)

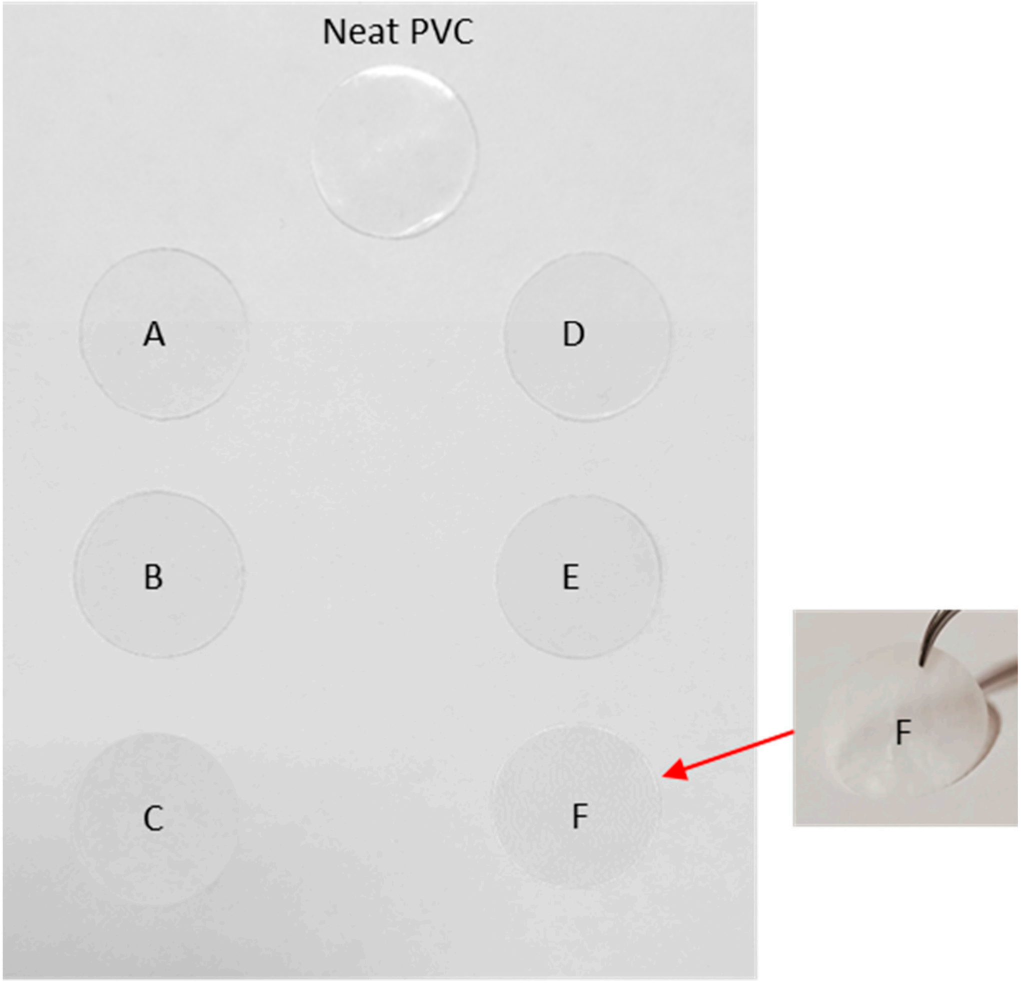

Figure S3. Image of neat PVC and PVC blend films loaded with 5 wt% concentration of C12mimDMSIP (**A**), C14mimDMSIP (**B**), C16mimDMSIP (**C**) and C12mimBF_4 (**D**), C14mimBF_4 (**E**), C16mimBF_4 (**F**). The PVC/5%C16mimBF_4 blend film was slightly whitish and opaque (**F**)

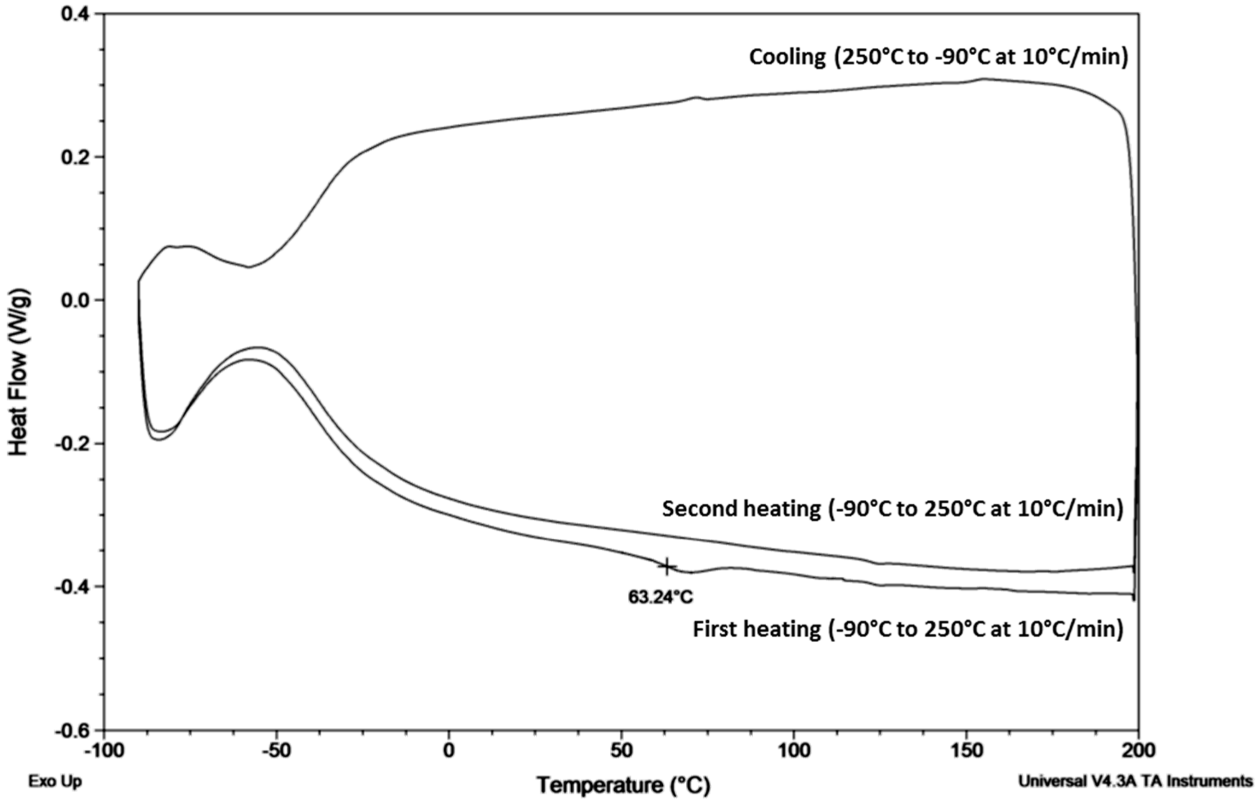

Figure S4. DSC curves of neat PVC TOTM.

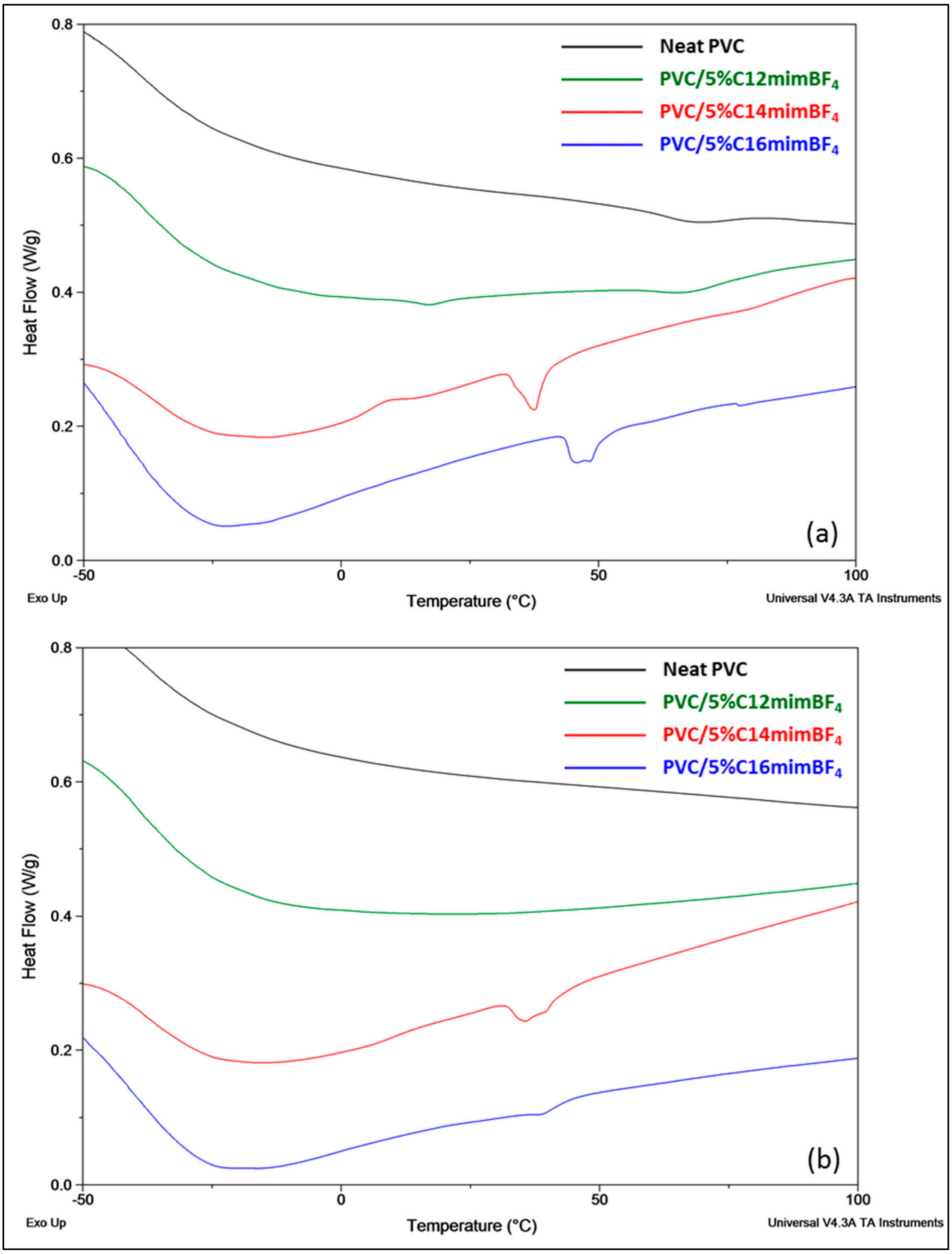

Figure S5. DSC curves of neat PVC TOTM and PVC/C*n*mimBF4 blend films (n = 12, 14, 16). (**a**) First heating, (**b**) second heating. Curves are displaced for clarity.

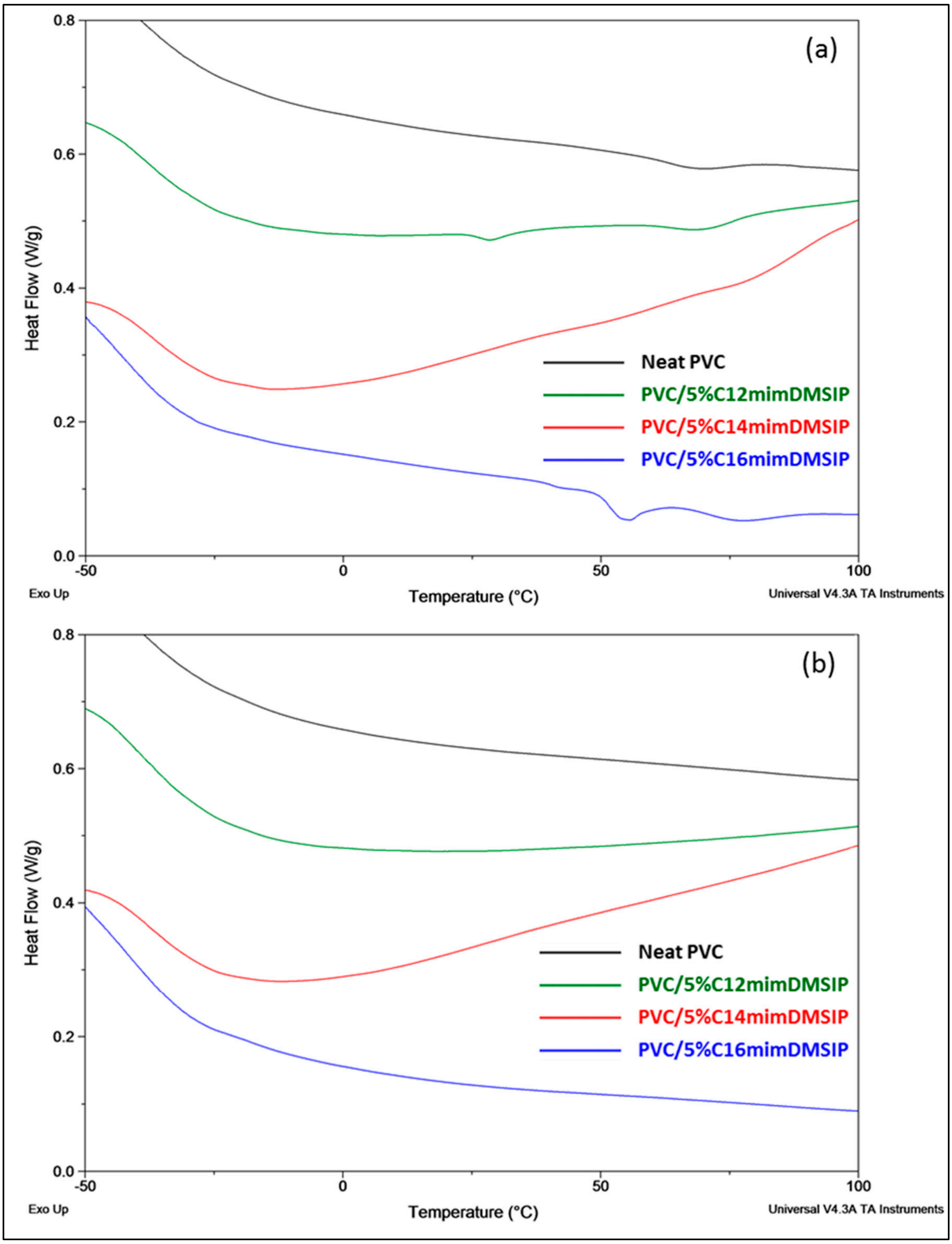

Figure S6. DSC curves of neat PVC TOTM and PVC/C*n*mimDMSIP blend films (n = 12, 14, 16). (**a**) First heating, (**b**) second heating. Curves are displaced for clarity.

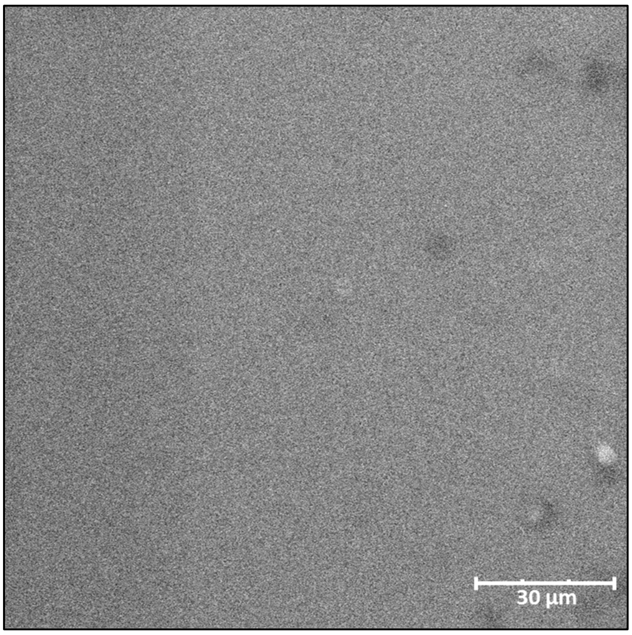

Figure S7. SEM picture (magnification 2000×) of neat PVC

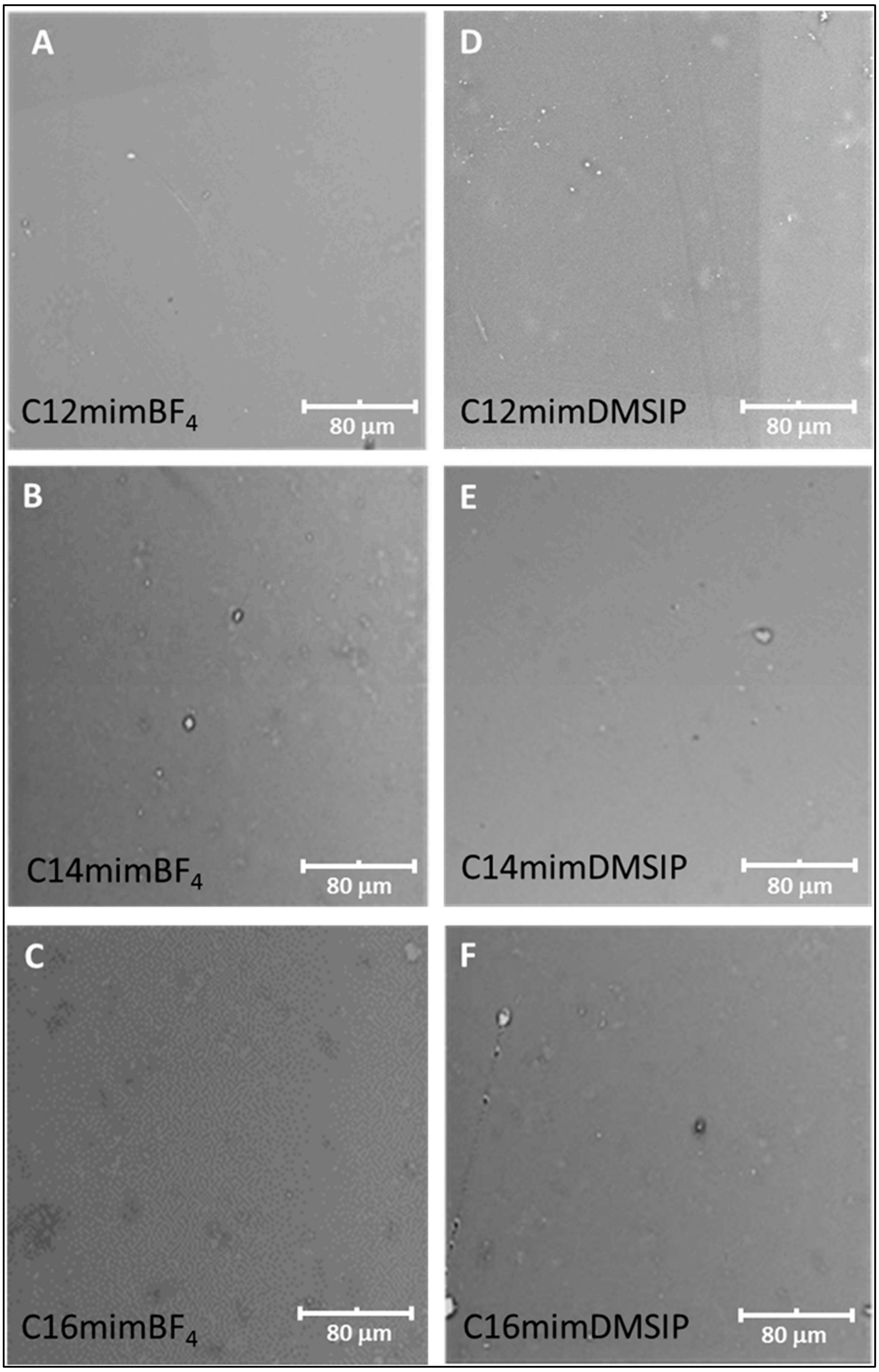

Figure S8. SEM pictures (magnification 1000×) of PVC blend films containing the 0.5 wt% concentration of (**A**) C12mimBF4, (**B**) C14mimBF4, (**C**) C16mimBF4, (**D**) C12mimDMSIP, (**E**) C14mimDMSIP, (**F**) C16mimDMSIP.

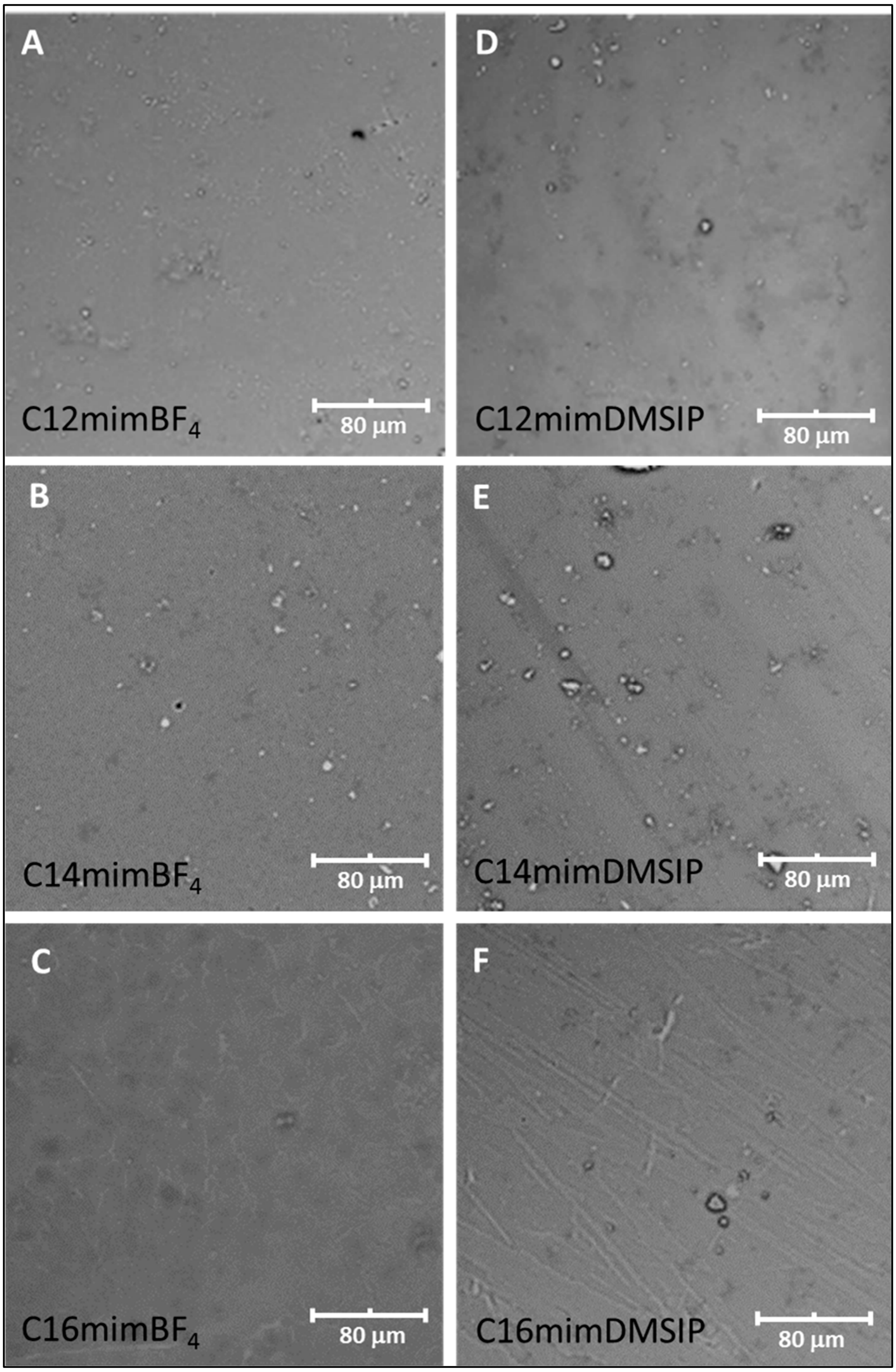

Figure S9. SEM pictures (magnification 1000×) of PVC blend films containing the 1 wt% concentration of (**A**) C12mimBF4, (**B**) C14mimBF4, (**C**) C16mimBF4, (**D**) C12mimDMSIP, (**E**) C14mimDMSIP, (**F**) C16mimDMSIP.

Bibliography

[1] Holbrey, J. D.; Reichert, W. M.; Swatloski, R. P.; Broker, G. A.; Pitner, W. R.; Seddon, K. R.; Rogers, R. D. Efficient, halide free synthesis of new, low cost ionic liquids: 1, 3-dialkylimidazolium salts containing methyl-and ethyl-sulfate anions. *Green Chem.* **2002**, 4(5), 407-413. https://doi.org/10.1039/B204469B.

[2] Colonna, M.; Berti, C.; Binassi, E.; Fiorini, M.; Sullalti, S.; Acquasanta, F.; Vannini, M.; Di Gioia, D.; Aloisio, I. Imidazolium poly(butylene terephthalate) ionomers with long-term antimicrobial activity. *Polymer* **2012**, 53, 1823–1830. https://doi.org/10.1016/j.polymer.2012.03.003.

[3] Bakulina, O. D.; Ivanov, M. Y.; Prikhod'ko, S. A.; Pylaeva, S.; Zaytseva, I. V.; Surovtsev, N. V.; Adonin, N.Y.; Fedin, M. V. Nanocage formation and structural anomalies in imidazolium ionic liquid glasses governed by alkyl chains of cations. *Nanoscale* **2020**, 12 (38), 19982-19991. https://doi.org/10.1039/D0NR06065H.
